# Self‐Report Tool for Identification of Individuals With Coronary Atherosclerosis: The Swedish CardioPulmonary BioImage Study

**DOI:** 10.1161/JAHA.124.034603

**Published:** 2024-07-03

**Authors:** Göran Bergström, Eva Hagberg, Elias Björnson, Martin Adiels, Carl Bonander, Ulf Strömberg, Jonas Andersson, Mattias Brunström, Carl‐Johan Carlhäll, Gunnar Engström, David Erlinge, Isabel Goncalves, Anders Gummesson, Emil Hagström, Ola Hjelmgren, Stefan James, Magnus Janzon, Lena Jonasson, Lars Lind, Martin Magnusson, Viktor Oskarsson, Johan Sundström, Per Svensson, Stefan Söderberg, Raquel Themudo, Carl Johan Östgren, Tomas Jernberg

**Affiliations:** ^1^ Department of Molecular and Clinical Medicine Institute of Medicine, Sahlgrenska Academy, University of Gothenburg Gothenburg Sweden; ^2^ Department of Clinical Physiology Region Västra Götaland, Sahlgrenska University Hospital Gothenburg Sweden; ^3^ School of Public Health and Community Medicine Institute of Medicine, University of Gothenburg Gothenburg Sweden; ^4^ Centre for Societal Risk Research Karlstad University Karlstad Sweden; ^5^ Department of Research and Development Region Halland Halmstad Sweden; ^6^ Department of Public Health and Clinical Medicine Umeå University Umeå Sweden; ^7^ Center for Medical Image Science and Visualization (CMIV) Linköping University Linköping Sweden; ^8^ Department of Clinical Physiology in Linköping, Department of Health, Medicine and Caring Sciences Linköping University Linköping Sweden; ^9^ Department of Clinical Sciences in Malmö Lund University Malmö Sweden; ^10^ Department of Clinical Sciences Lund, Cardiology Lund University, Skåne University Hospital Lund Sweden; ^11^ Department of Cardiology Skåne University Hospital Malmö Sweden; ^12^ Cardiovascular Research Translational Studies, Department of Clinical Sciences Malmö Lund University Malmö Sweden; ^13^ Department of Clinical Genetics and Genomics Sahlgrenska University Hospital Gothenburg Sweden; ^14^ Department of Medical Sciences Cardiology, Uppsala University Uppsala Sweden; ^15^ Uppsala Clinical Research Center Uppsala University Uppsala Sweden; ^16^ Pediatric Heart Centre, Queen Silvias Childrens hospital Sahlgrenska University Hospital Gothenburg Sweden; ^17^ Department of Cardiology and Department of Health, Medicine and Caring Sciences, Unit of Cardiovascular Sciences Linköping University Linköping Sweden; ^18^ Department of Medical Sciences, Clinical Epidemiology Uppsala University Uppsala Sweden; ^19^ North‐West University Potchefstroom South Africa; ^20^ Wallenberg Center for Molecular Medicine Lund University Lund Sweden; ^21^ Piteå Research Unit Region Norrbotten Piteå Sweden; ^22^ Department of Medical Sciences Uppsala University Uppsala Sweden; ^23^ The George Institute for Global Health University of New South Wales Sydney New South Wales Australia; ^24^ Department of Clinical Science and Education, Södersjukhuset Karolinska Institutet Stockholm Sweden; ^25^ Department of Cardiology Södersjukhuset Stockholm Sweden; ^26^ Department of Clinical Science, Intervention and Technology, Division of Medical Imaging and Technology Karolinska Institute Stockholm Sweden; ^27^ Department of Radiology Karolinska University Hospital in Huddinge Stockholm Sweden; ^28^ Department of Health, Medicine and Caring Sciences Linköping University Linköping Sweden; ^29^ Department of Clinical Sciences Danderyd University Hospital, Karolinska Institutet Stockholm Sweden

**Keywords:** coronary artery calcium score, coronary atherosclerosis, risk prediction tool, segment involvement score, self‐reported data, Cardiovascular Disease, Epidemiology, Primary Prevention

## Abstract

**Background:**

Coronary atherosclerosis detected by imaging is a marker of elevated cardiovascular risk. However, imaging involves large resources and exposure to radiation. The aim was, therefore, to test whether nonimaging data, specifically data that can be self‐reported, could be used to identify individuals with moderate to severe coronary atherosclerosis.

**Methods and Results:**

We used data from the population‐based SCAPIS (Swedish CardioPulmonary BioImage Study) in individuals with coronary computed tomography angiography (n=25 182) and coronary artery calcification score (n=28 701), aged 50 to 64 years without previous ischemic heart disease. We developed a risk prediction tool using variables that could be assessed from home (self‐report tool). For comparison, we also developed a tool using variables from laboratory tests, physical examinations, and self‐report (clinical tool) and evaluated both models using receiver operating characteristic curve analysis, external validation, and benchmarked against factors in the pooled cohort equation. The self‐report tool (n=14 variables) and the clinical tool (n=23 variables) showed high‐to‐excellent discriminative ability to identify a segment involvement score ≥4 (area under the curve 0.79 and 0.80, respectively) and significantly better than the pooled cohort equation (area under the curve 0.76, *P*<0.001). The tools showed a larger net benefit in clinical decision‐making at relevant threshold probabilities. The self‐report tool identified 65% of all individuals with a segment involvement score ≥4 in the top 30% of the highest‐risk individuals. Tools developed for coronary artery calcification score ≥100 performed similarly.

**Conclusions:**

We have developed a self‐report tool that effectively identifies individuals with moderate to severe coronary atherosclerosis. The self‐report tool may serve as prescreening tool toward a cost‐effective computed tomography‐based screening program for high‐risk individuals.

**Nonstandard Abbreviations and Acronyms**
CACS: coronary artery calcification score
PCE: pooled cohort equation
SCAPIS: Swedish CardioPulmonary BioImage Study
SHAP: Shapley Additive Explanations
SIS: segment involvement score

**Clinical Perspective**

**What Is New?**

- We have developed a self‐report tool with a good‐to‐excellent discriminative ability that identifies individuals with moderate to severe coronary atherosclerosis.
- The self‐report tool can be executed from home and has a similar performance to a clinical tool requiring a clinical visit involving blood tests and physical examination.

**What Are the Clinical Implications?**

- The self‐report tool could serve as an initial step toward a cost‐effective screening program to identify high‐risk individuals or used to identify individuals who would benefit from further risk refinement by cardiac imaging.

Asymptomatic individuals with signs of coronary atherosclerosis on imaging are considered to be at high risk of future ischemic heart disease (IHD).^1^, ^2^, ^3^ A coronary artery calcification score (CACS) of ≥100, derived from computed tomography (CT) imaging, suggests benefits of statin therapy regardless of low‐density lipoprotein concentrations in individuals with an intermediate IHD risk, according to the pooled cohort equation (PCE) risk calculator (7.5%–20.0%).^1^ Imaging with coronary CT angiography (CCTA) holds an even bigger promise, because it also visualizes noncalcified coronary atherosclerosis, degree of stenosis, and plaque characteristics, factors that are directly related to an increased risk of future clinical events^4^, ^5^, ^6^, ^7^, ^8^ and that improve risk prediction beyond clinical risk scores such as the PCE.^8^, ^9^, ^10^ However, the drawbacks of imaging include limited availability, high costs, and risks associated with radiation and use of contrast agents. Therefore, it is of great interest to use nonimaging data to develop tools that identify individuals with high risk of coronary atherosclerosis. These tools could be directly used to identify individuals with an increased risk of IHD or to identify individuals in whom imaging could do more benefit than harm.

To facilitate participation and to reduce costs, it would be advantageous if the tools could be easily administered and based on self‐reported data, not requiring a visit to a health care center. A few recent studies show promising results in the self‐reported assessment of cardiovascular risk.^11^, ^12^

The aim of this study was to test whether the nonimaging data collected in the SCAPIS (Swedish CardioPulmonary BioImage Study)^13^, ^14^ could be used to identify individuals with moderate to severe coronary atherosclerosis (defined by the Coronary Artery Disease Reporting and Data System criteria^15^ as segment involvement score [SIS] ≥4 or CACS ≥100). These cutoff values were selected because previous work with CT^2^, ^16^ and CCTA^7^, ^8^, ^17^, ^18^, ^19^ have consistently shown that the risk of future IHD is markedly increased at this level of coronary atherosclerosis. The more specific aim was to test if a tool exclusively based on self‐reported data could be equally effective as a tool based on the combination of self‐reported data and clinical data. The tools were developed and tested in SCAPIS and externally validated in a separate cohort. We included only individuals without previous coronary heart disease and tools were benchmarked against the PCE risk calculator.

## METHODS

### The SCAPIS Data Set

SCAPIS (n=30 154) is a population‐based multicenter cohort study of randomly selected men and women aged 50 to 64 years, mainly of European ancestry.^14^ The data collection was performed between 2014 and 2018 at 6 Swedish university hospitals. A comprehensive examination protocol was used, including cardiac imaging, physical examinations, routine laboratory tests, and questionnaires.^13^, ^14^ SCAPIS was approved by the ethics committee in Umeå, Sweden (number 2010–228‐31M), all participants gave written informed consent, and the study protocols adhered to the Declaration of Helsinki. Due to the nature of the sensitive personal data and study materials they cannot be made freely available. However, by contacting the corresponding author or the study organization (www.scapis.org), procedures for sharing data, analytic methods, and study materials for reproducing the results or replicating the procedure can be arranged following Swedish legislation.

The data set used for external validation (n=1111) was the single‐site SCAPIS pilot trial collected in 2012. This study's primary objective was to provide insights into the feasibility of performing the nationwide SCAPIS and we therefore used a stratified selection of individuals aimed at low and high socioeconomic areas within the city of Gothenburg.^20^ Participants are unique from the nationwide SCAPIS and different equipment and different staff were used.

### Study Populations

Participants without previous coronary heart disease (myocardial infarction or coronary revascularization) and with high‐quality imaging of their coronary arteries using CCTA (n=25 182) or high‐quality CT imaging for CAC (n=28 701) were selected to address the primary aim of the study (see the following section for imaging details). Similarly, individuals from the SCAPIS pilot trial (n=872 with CCTA and n=1062 with CT imaging for CACS) were used for validation.

### Cardiac Image Acquisition and Analyses

As previously described,^14^ we performed cardiac CT scanning with and without contrast agent using a dedicated dual‐source CT scanner equipped with a Stellar Detector (Somatom Definition Flash, Siemens Medical Solution, Forchheim, Germany). CCTA scans were read using the syngo.via software, and the 18‐coronary segment model defined by the Society of Cardiovascular Computed Tomography was used to report coronary atherosclerosis as outlined in Data S1. SIS was calculated as the sum of all coronary artery segments with atherosclerosis.^8^ CACS was analyzed using the method by Agatston.^21^

### Factors Used for Developing the Prediction Tools

All factors used to develop the prediction tools are summarized in Table S1. Details on how they were collected can be found elsewhere.^13^, ^14^ Information on self‐reported health, family history, medication, occupational and environmental exposure, lifestyle, psychosocial well‐being, socioeconomic status, and other social determinants was collected from questionnaires administered at the clinical visit. The questionnaire was filled in by the participants themselves with minimal interaction by staff members. Data from electronic health records were not used but the participants were asked to bring their prescriptions to the site visit. Biochemistry analyses were analyzed from venous blood samples collected after an overnight fast. Height, weight, and hip and waist circumference were measured by trained staff. Physical activity was assessed using an accelerometer. Blood pressure was measured with an automatic device (Omron M10‐IT, Omron Health Care Co, Kyoto, Japan). Lung function was measured using spirometry after bronchodilatation. Variables were used in their original scale (not normalized) during model training.

### Outcomes

We used 2 different outcome variables: SIS ≥4 and CACS ≥100. A detailed rationale for the selected cutoff values is presented in Data S1. According to recent consensus by Coronary Artery Disease Reporting and Data System,^15^ SIS ≥4 and CACS ≥100 represent moderate to severe coronary atherosclerosis.

### Statistical Analysis

In this study, we developed a self‐report tool based on all available self‐reported data. Additionally, a clinical tool was developed, using all SCAPIS data, including self‐reported data, blood tests, and physical examinations. The purpose of the clinical tool was to serve as a comparative measure to the self‐report model. From the SCAPIS baseline data, we identified 105 factors as potential predictors in the self‐report tool. A total of 127 factors was identified as potential predictors in the clinical tool (all factors are summarized in Table S1). Factors in the self‐report tool are possible to report without visiting a health care facility. The performance of both of the tools was bench‐marked against the 10‐year risk of atherosclerotic cardiovascular disease according to the PCE.^1^ As a sensitivity analyses we also tested the performance of the European Systematic Coronary Risk Evaluation 2.^22^ We also estimated the average time it took for 10 coauthors to fill out the self‐report tool when presented in paper format.

Descriptive data are presented without imputations, and data used for the prediction tools were imputed using the K‐nearest neighbor algorithm with 5 neighbors. For descriptive data, numbers, percentages, mean values (SD), and median values (interquartile range) were calculated. A grid‐search was employed in order to optimize model hyperparameters. We followed the Transparent Reporting of a Multivariable Prediction Model (TRIPOD) for Individual Prognosis or Diagnosis statement for transparent reporting.^23^ All analyses were performed using R version 4.1.3.

#### Data Reduction

To identify and include the most relevant factors in our tools, we performed data reduction using a combination of data‐driven techniques (Boruta) and manual techniques (described in detail in Data S1). In a sensitivity analyses, we also tested a model based on all 127 variables without prior data selection.

#### Development of Assessment Tools for Moderate to Severe Coronary Atherosclerosis

After the data reduction, we used XGBoost (Extreme Gradient Boosting; a decision‐tree‐based machine learning method) to develop the tools to identify SIS ≥4 and CACS ≥100. The data set was randomly split into a 75% training set and a 25% test set. The area under the receiver operating characteristic curve (area under the curve [AUC]) was calculated and validated in the external study population and compared using the DeLong test.^24^ We avoided overfitting by 3 strategies. First, hyperparameter tuning was employed to minimize the risk of overfitting; second, we included an internal‐validation subset of the data that the model had never trained on; and third, we used a separate cohort as a validation cohort. Our results show that the models appear correctly trained. Calibration curves were constructed and compared between internal and external validation. We also calculated the importance ranking of each variable with XGBoost and the Shapley Additive Explanations (SHAP)‐value. SHAP values represent the relationship between each factor and the outcome for each subject, for which a higher SHAP value confers a higher assessed risk by the XGBoost algorithm.

#### Methodological Considerations

Machine‐learning methodology was preferred over logistic regression modeling for several reasons. First, the model can account for complex variable relationships with the outcome and interactions between variables without the need for explicit specification of this. Second, tree‐based models do not rely on the estimation of coefficients for each independent variable and therefore the impact of multicollinearity is generally minimal. Third, model interpretability is improved with possibility of both overall and per‐subject variable importance analysis. Fourth, variable selection procedure is simpler and more accurate.

#### Stratification for Age, Sex, and Socioeconomic Status

Analyses were stratified according to sex and age (50–54, 55–59, and 60–64 years). In addition, to examine the impact of socioeconomic factors, analyses were stratified by the individuals' socioeconomic status based on 3 different characteristics: education (university degree or lower education), country of birth (born in Sweden or not), and the ability to raise a sum of 20 000 Swedish kronor within a week (equivalent to US$1900).

#### Construction of Population‐Ordered Distributions and Decision Curves

To assess our tools' performance in a potential screening situation, we constructed population‐ordered distribution tables with 10 groups of equal size (deciles). We also constructed clinical decision curves to evaluate if our tools gave a net benefit over the PCE at relevant threshold probabilities.^25^

## RESULTS

### Study Population

**Figure 1:**
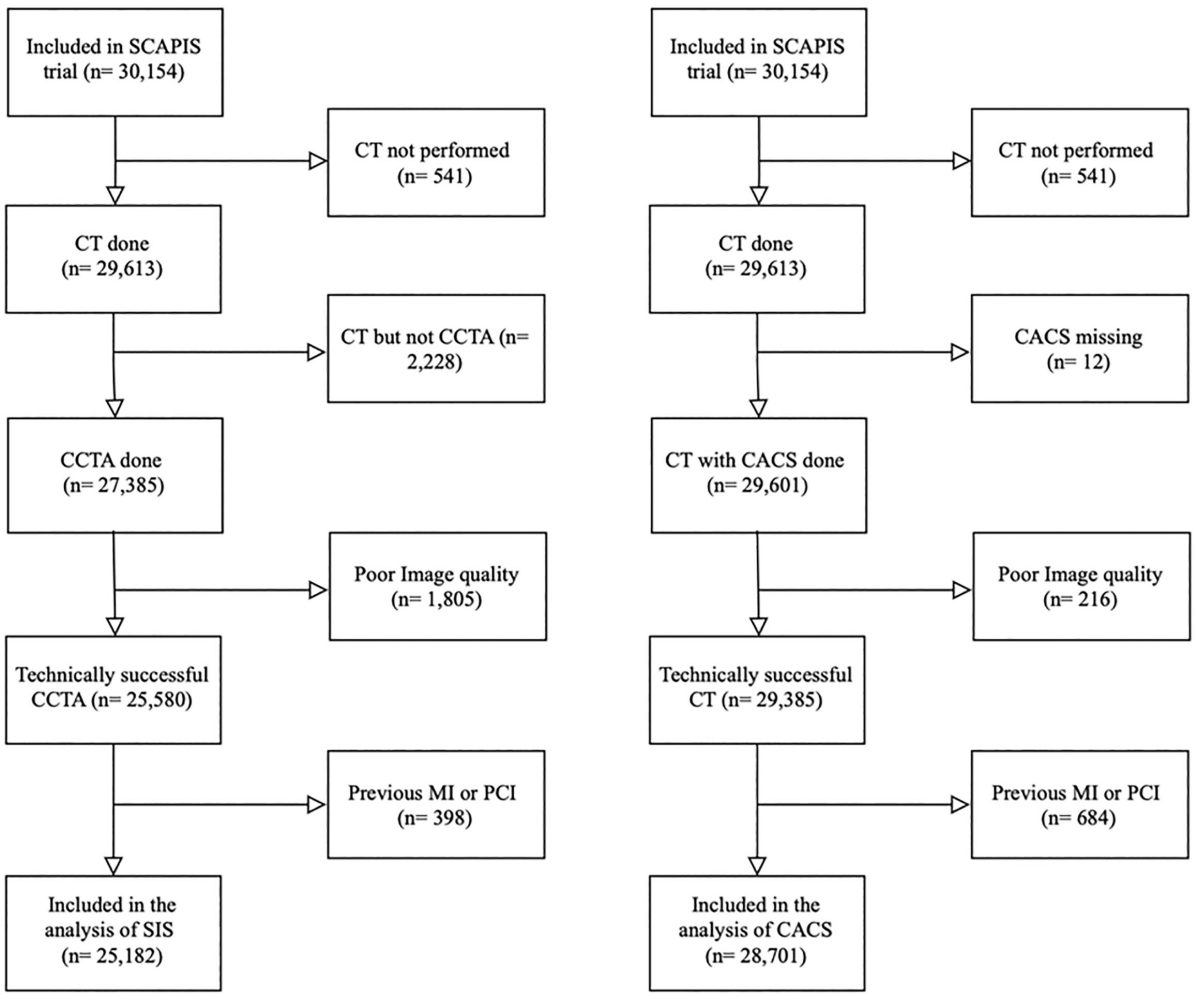
Flow diagram of study inclusion. CACS indicates coronary artery calcification score; CCTA, coronary computed tomography angiography; CT, computed tomography; MI, myocardial infarction; PCI, percutaneous coronary intervention; SCAPIS, Swedish CardioPulmonary BioImage Study; and SIS, segment involvement score.

The study inclusion is shown in Figure 1. In total, 25 182 individuals were included in the cohort studying SIS ≥4 as an outcome, for whom the characteristics are shown in Table 1. In total, 11.9% of the participants had SIS ≥4 (5.1% of women and 19% of men). In general, participants with SIS ≥4 had a more severe risk factor profile and their 10‐year risk of atherosclerotic cardiovascular disease (according to the PCE) was more than 50% higher than participants with SIS <4 (1.8 times higher in women and 1.5 times in men). In total, 28 701 individuals were included in the cohort that studied CACS ≥100 as an outcome and their characteristics are shown in Table S2. In total, 12.0% of the participants had CACS ≥100 (6.1% of women and 22.9% of men).

**Table 1:** Clinical Characteristics of Individuals (n=25 182) With High‐Quality Coronary Computed Tomography Angiography, Without Known Coronary Heart Disease, Stratified By Sex and Segment Involvement Score (SIS) ≥4

	Total	Female sex		Male sex	
	All SIS	SIS<4	SIS≥4	SIS<4	SIS≥4
No.	25 182	12 094	644	10 085	2359
Socio demographics					
Age, y	57.4±4.3	57.3±4.3	59.6±3.9	56.9±4.3	59.3±4.1
Education, university degree, n (%)	11 300 (46.0)	6010 (49.7)	242 (37.6)	4183 (41.5)	865 (36.7)
Born in Sweden, yes, n (%)	20 893 (83.0)	10 030 (82.9)	533 (82.8)	8455 (83.8)	1875 (79.5)
Can raise 20 000 Swedish krona* within a week, yes, n (%)	22 515 (89.4)	10 748 (88.9)	551 (85.6)	9152 (90.7)	2064 (87.5)
Anthropometry					
Weight at age 20, kg	66.5±11.3	58.0±7.8	58.8±9.0	73.0±8.9	73.6±9.5
Weight, kg	80.1±15.5	72.2±13.0	74.6±14.6	87.6±13.3	89.9±14.5
Height, cm	173±9.6	166±6.4	165±6.1	180±6.9	179±7.0
Waist circumference, cm	94.0±12.6	88.7±11.9	93.1±13.2	98.6±10.7	101.9±11.4
Hip circumference, cm	102±8.5	103±9.7	104±10.8	102±6.9	102±7.4
Smoking status					
Current smoker, n (%)	3215 (12.8)	1504 (12.4)	172 (26.7)	1142 (11.3)	397 (16.8)
Mean pack‐years, y	7.2±11.7	6.8±10.6	15.9±15.6	6.4±11.5	10.5±14.7
Duration of smoking, y	11.6±15.2	11.8±15.3	22.9±17.9	9.8±14.6	15.1±17.0
Treatment					
Cholesterol‐lowering medication, n (%)	1624 (6.4)	534 (4.4)	117 (18.2)	578 (5.7)	395 (16.7)
Antihypertensive medication, n (%)	4437 (17.6)	1852 (15.3)	233 (36.2)	1554 (15.4)	798 (33.8)
Diabetes medication, n (%)	717 (2.8)	198 (1.6)	49 (7.6)	273 (2.7)	197 (8.4)
Diagnosis					
Diagnosed hypertension, n (%)	2056 (8.2)	807 (6.7)	115 (17.9)	707 (7.0)	427 (18.1)
Diabetes duration, age, y	0.3 (2.6)	0.2 (2.3)	1.2 (6.0)	0.3 (2.1)	0.9 (4.2)
Blood pressure, mm Hg					
Systolic, mm Hg	126±17	122±18	130±18	128±15	133±16
Diastolic, mm Hg	78±11	77±11	79±11	78±10	80±10
Clinical chemistry					
Total cholesterol, mmol/L	5.5±1.0	5.7±1.0	5.8±1.2	5.4±1.0	5.5±1.1
High‐density lipoprotein cholesterol, mmol/L	1.6±0.5	1.9±0.5	1.7±0.5	1.4±0.4	1.4±0.4
Low‐density lipoprotein, calculated, cholesterol, mmol/L	3.5±0.9	3.4±0.9	3.7±1.1	3.5±0.9	3.6±1.1
Triglycerides, mmol/L	1.2±0.8	1.1±0.6	1.4±0.8	1.3±0.9	1.5±0.9
Glucose, mmol/mL	5.7±1.0	5.5±0.8	5.9±1.4	5.8±1.0	6.2±1.5
Glycated hemoglobin, mmol/mL	36.3±5.9	35.9±4.9	38.6±8.0	36.0±5.7	38.4±8.8
High‐sensitive C‐reactive protein, mg/L	2.1±3.9	2.1±3.6	2.7±5.2	1.9±3.7	2.4±5.4
Risk score					
Pooled cohort equation, %	6.2±5.5	3.2±2.7	5.9±4.3	8.4±5.3	12.5±6.9
Heredity					
Family history of premature myocardial infarction, n (%)	1619 (6.4)	821 (6.8)	79 (12.3)	515 (5.1)	204 (8.6)
Family history of premature stroke, n (%)	1468 (5.8)	780 (6.4)	56 (8.7)	469 (4.7)	163 (6.9)
Symptoms					
Angina like symptoms†, %	381 (1.5)	209 (1.7)	18 (2.8)	103 (1.0)	51 (2.2)

SIS indicates segment involvement score.

* Equivalent to US$ 1900.

^†^ According to the Rose questionnaire.

In the SCAPIS cohorts studying SIS ≥4 and CACS ≥100 as an outcome there were a few individuals with self‐reported symptoms resembling those of angina, as per the Rose criteria,^26^ 1.5% and 1.6% respectively. There was a significantly higher prevalence of these symptoms observed in the high‐risk groups versus low‐risk groups (Table 1 and Table S2).

In the validation cohort, a higher proportion of participants born outside Sweden and a lower proportion with university education were observed compared with the SCAPIS data set. Details on study inclusion and characteristics of the validation cohorts are shown in Figure S1, Tables S3 and S4. In the validation cohort, angina like symptoms could not be reported due to technical errors in administration of the Rose questionnaire.

### Factors Selected for Developing the Self‐Report and Clinical Prediction Tools

A summary of missing data for each available factor and specification of the relevant factors identified by the Boruta algorithm is shown in Table S1. A description of all individual factors can be found at www.scapis.org/portal/variables. Following discussion among lead authors, the self‐report tool incorporated 14 factors (Table 2). These factors included 7 on demographic and anthropometric information (sex, age, body weight at age 20, body weight, body height, waist circumference, hip circumference), 2 on smoking status (cigarette pack‐years and smoking duration), 4 on existing health conditions (lipid‐lowering medication, antihypertensive medication, diabetes duration, and diagnosed hypertension), and 1 on family history (heredity for myocardial infarction). The clinical tool incorporated 23 factors (Table 2), with all the 14 factors in the self‐report model included but also adding vital signs (3 factors) and laboratory measurements (6 factors). Additional details on data selection can be found in Data S1. It took 5 to 8 minutes to fill out the self‐report questionnaire when tested on 10 coauthors.

**Table 2:** Factors in the Self‐Report, Clinical Tool and the Pooled Cohort Equation and Their Measurement Scales

Category	Factor	Self‐report tool (scale)	Clinical tool (scale)	PCE (scale)
Demographics	Sex	Binary	Binary	Binary
	Age	Continuous	Continuous	Continuous
	Race	N/A*	N/A	Categorical
Anthropometry	Body weight	Continuous	Continuous	N/A
	Body weight at age 20	Continuous	Continuous	N/A
	Body height	Continuous	Continuous	N/A
	Waist circumference	Continuous	Continuous	N/A
	Hip circumference	Continuous	Continuous	N/A
Smoking status	Cigarette pack‐years	Continuous	Continuous	N/A
	Smoking duration	Continuous	Continuous	N/A
	Currently smoking	N/A	N/A	Binary
Health condition	Lipid‐lowering medication	Binary	Binary	Binary
	Antihypertensive medication	Binary	Binary	Binary
	Diagnosed hypertension	Binary	Binary	Binary
	Diabetes duration	Continuous	Continuous	Binary
	Diagnosed diabetes	N/A	N/A	Binary
Blood tests	Glycated hemoglobin	N/A	Continuous	Continuous
	Total cholesterol	N/A	Continuous	Continuous
	Plasma glucose	N/A	Continuous	N/A
	Creatinine	N/A	Continuous	N/A
	Plasma triglycerides	N/A	Continuous	N/A
	High‐density lipoprotein cholesterol	N/A	Continuous	Continuous
Clinical assessment	Systolic blood pressure	N/A	Continuous	Continuous
	Heart rate	N/A	Continuous	N/A
	Diastolic blood pressure	N/A	Continuous	N/A
Family history	Heredity for myocardial infarction	Binary	Binary	N/A

N/A indicates not applicable and PCE, pooled cohort equation.

### Prediction Tools

**Figure 2:**
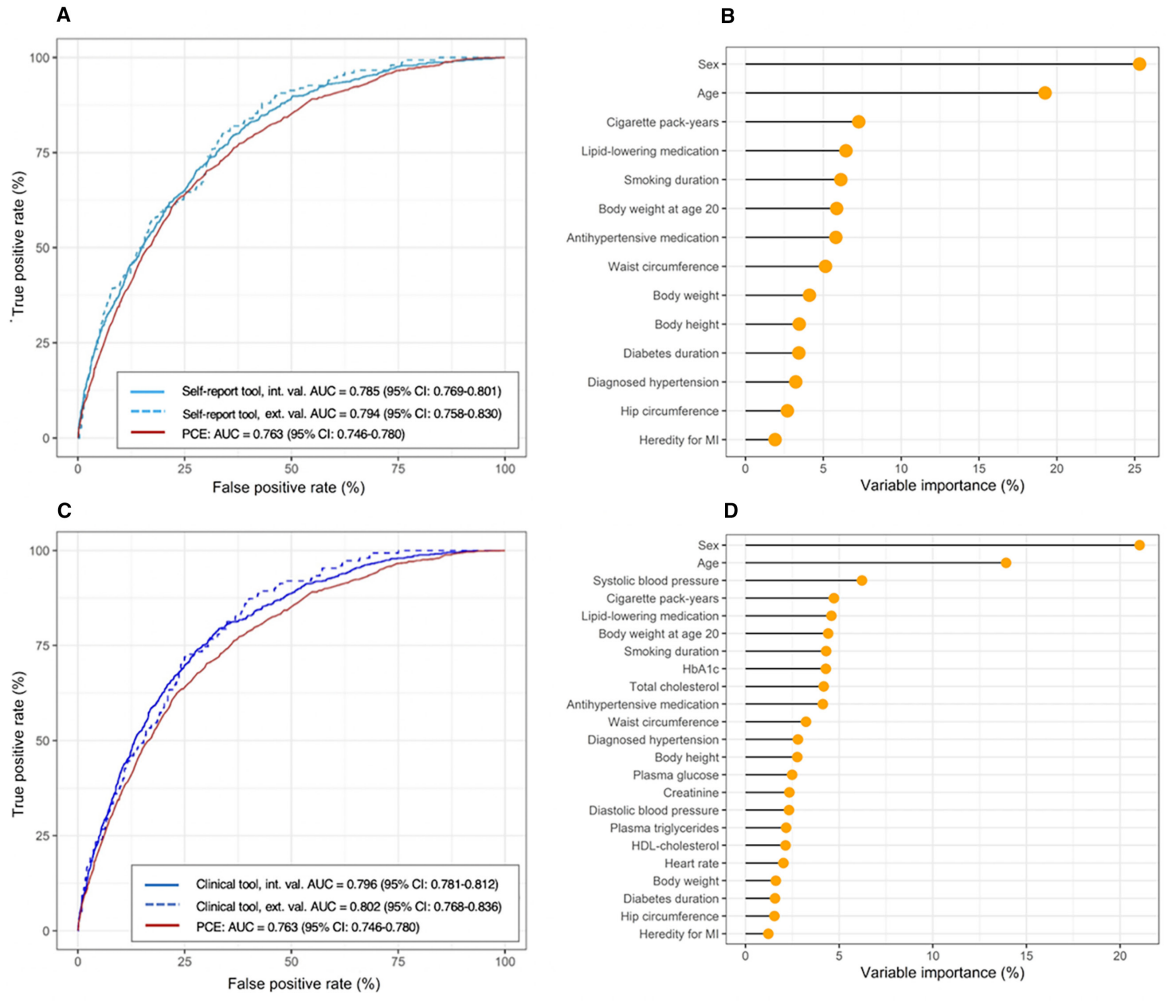
Receiver operating characteristic curve for the self‐report tool's assessment of SIS≥4 in the internal and external validation group compared with PCE. **A,** Self‐report tool vs PCE, *P*<0.001, *P*<0.001 for internal and external validation respectively. Variable importance of the self‐ report tool (**B**). ROC curve for the clinical tool's assessment of SIS≥4 compared with PCE (**C**, clinical tool vs PCE, *P*<0.001, *P*<0.001 for internal and external validation respectively). Variable importance of the clinical tool (**D**). The DeLong test was used for statistical comparison. AUC indicates area under the curve; HbA1c, glycated hemoglobin; HDL, high‐density lipoprotein; MI, myocardial infarction; PCE, pooled cohort equation; and ROC, receiver operating characteristic.

The self‐report tool had a high‐to‐excellent discriminatory capacity for SIS ≥4 in the external validation cohort (AUC, 0.79 [95% CI, 0.76–0.83]); and its discriminatory capacity was significantly better than the PCE risk score (AUC, 0.76 [95% CI, 0.75–0.78], *P*<0.005, Figure 2A). With respect to variable importance of the self‐report tool, sex and age were the most important variables, followed by cigarette pack‐years, lipid‐lowering medication, smoking duration, and several anthropometric measures (Figure 2B). The clinical tool performed slightly better than the self‐report tool (AUC, 0.80 [95% CI, 0.77–0.84], *P*<0.05, Figure 2C). The variable importance of the clinical tool is presented in Figure 2D, where systolic blood pressure and laboratory measurements of glycated hemoglobin and total cholesterol also were important for prediction. Both tools were well calibrated (Figure S2). SHAP plots for the interpretation of each variable's contribution to the tools are presented in Figures S3 through S4. Results for the prediction of CACS ≥100 were largely similar (Figures S5 through S8).

### Sensitivity and Stratification Analyses

The European Systematic Coronary Risk Evaluation 2 algorithm did not outperform the PCE risk algorithm in identifying either SIS ≥4 (AUC, 0.75 [95% CI, 0.73–0.77]) or CACS ≥100 (AUC, 0.74, [95% CI, 0.72–0.76]).

**Figure 3:**
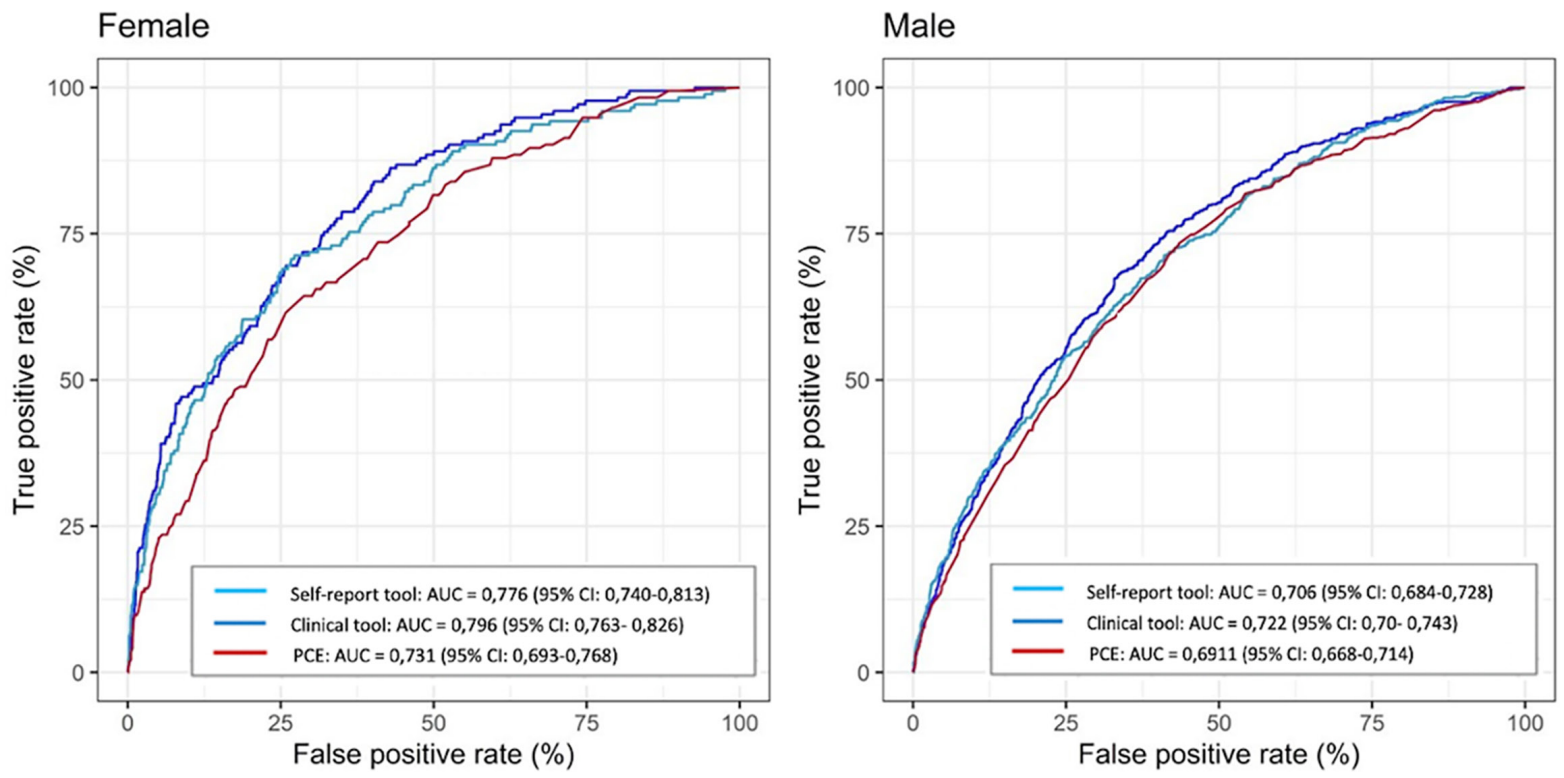
Receiver operating characteristic curves for the self‐report tool and the clinical tool assessing SIS≥4 vs PCE, stratified by sex. Female; self‐report tool vs PCE (*P*<0.001), clinical tool vs PCE (*P*<0.001). Male; self‐report tool vs PCE (*P*<0.001), clinical tool vs PCE (NS). The DeLong test was used for statistical comparison. AUC, indicates area under the curve; ns, nonsignificant; PCE, pooled cohort equation; and SIS, segment involvement score.

**Figure 4:**
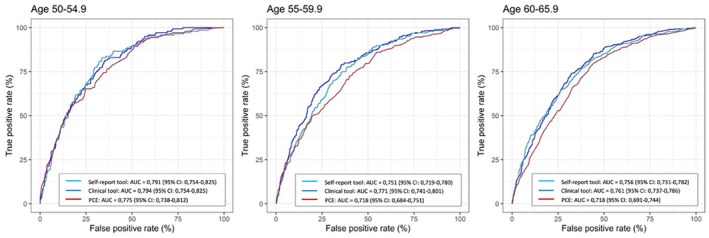
Receiver operating characteristic curves for the self‐report tool and the clinical tool assessing SIS≥4 vs PCE, stratified by age. Age 50 to 54.9 (NS, NS for self‐report and clinical tool respectively), 55 to 59.9 (*P*<0.05, *P*<0.001 for self‐report and clinical tool respectively), 60–65.9 (*P*<0.001, *P*<0.001 for self‐report and clinical tool respectively). The DeLong test was used for statistical comparison. AUC, indicates area under the curve; NS, nonsignificant; PCE, pooled cohort equation; and SIS, segment involvement score.

In general, both prediction tools performed better in women than in men (Figure 3); and they performed better in individuals older than 55 years of age (Figure 4). Similar results were seen for CACS (Figures S9 and S10). The self‐report tools were largely unaffected by stratification for education, country of birth, and financial resources (Tables S5 and S6). As an example, the AUC of the self‐ report tool was 0.79 (95% CI, 0.76–0.81) in university educated and 0.78 (95% CI, 0.76–0.78) in nonuniversity educated. The tool that was developed based on all 127 factors had an AUC of 0.80 (95% CI, 0.78–0.81).

### Population‐Ordered Distribution and Decision Curves

To exemplify how the prediction tools can be applied in a screening situation, we divided the population into 10 groups of equal size, ordered by predicted risk (Table 3). Upon visual inspection, we identified a high‐risk group in the individuals with the top 30% of assessed risk (ie, top 3 deciles), with a mean absolute risk of SIS ≥4 of 27.2% for the self‐report tool (Table 3). The middle 4 deciles suggested a moderate mean absolute risk, averaging 9.3%. Additionally, a low‐risk group was identified in the individuals in the bottom 30% of assessed risk, with a mean absolute risk of SIS ≥4 of 2.2. The clinical tool showed similar patterns (Table S7).

**Table 3:** Population‐Ordered Distribution for the Self‐Report Tool in Identifying Segment Involvement Score ≥4

Decile of risk	Cumulative % of population	Mean absolute risk for SIS≥4 (%)	Number of needed to image with CCTA per finding	Cumulative numbers of CCTAs per finding	Cumulative % of all positives	Age (years) *	Male (%)	SIS (number of segments)*	CACS (Agatston score)*	PCE (%)
1	10%	40.4	2.5	2.5	32.2	61.4	93.8	3.2	236.0	14.8
2	20%	23.8	4.2	3.1	51.2	60.2	92.7	2.1	101.0	11.5
3	30%	17.3	5.8	3.7	65.0	59.2	87.0	1.6	77.9	9.2
4	40%	15.3	6.6	4.1	77.2	57.0	78.5	1.5	65.1	7.3
5	50%	11.0	9.1	4.6	85.9	56.3	65.2	1.1	43.0	5.8
6	60%	7.0	14.3	5.2	91.5	55.9	54.1	0.8	24.6	4.6
7	70%	4.1	24.2	5.9	94.7	57.4	25.1	0,7	20.3	3.9
8	80%	4.0	25.2	6.5	97.9	57.5	5.4	0,6	14.6	3.0
9	90%	1.6	63.0	7.2	99.2	56.1	0.2	0,3	7.7	2.1
10	100%	1.1	90.0	8.0	100	53.1	0.0	0.3	6.42	1.2

The first decile presents individuals at the highest mean absolute risk. Top 3 deciles: high mean average risk (average risk 27.2%); Middle 4 deciles: moderate mean absolute risk (average risk 9.3%); Lower 3 deciles: low mean absolute risk (average risk 2.2%). CACS indicates coronary artery calcium score; CCTA, coronary computed tomography angiography; PCE, pooled cohort equation; and SIS, segment involvement score.

* Mean.

Among individuals who fell within the high‐risk group, we identified 64.6% and 67.3% of all individuals with SIS ≥4 using the self‐report and clinical tool, respectively. In order to find 1 individual with SIS ≥4 in the high‐risk group, an average of 3.7 CCTAs have to be performed for the self‐report tool and 3.5 CCTAs for the clinical tool. Similar results were observed for CACS ≥100 (Tables S8 and S9).

Decision curve analyses for SIS ≥4 and CACS ≥100 showed that both the self‐report and the clinical tool is superior to the PCE when applied to a population with a mean absolute risk corresponding to the high‐risk group (ie, 27%–28%) (Figures S11 and S12).

## DISCUSSION

This study shows that we can effectively use nonimaging data to identify individuals from the general population with a high likelihood of having moderate to severe coronary atherosclerosis. The discriminative ability to identify SIS ≥4 and CACS ≥100 was high‐to‐excellent in the external validation cohort (AUC, 0.79–0.81). Most important, the self‐report tool, based only on data that do not require a health care visit, performed almost equal to the clinical tool, which included blood tests and physical examinations.

The tools were developed in the SCAPIS population, which is a general population sample randomly selected from the people's registry with no exclusion criteria except language difficulties.^14^ For the current study we removed all participants with prior IHD to focus on a primary prevention population. However, the cohort was not strictly asymptomatic and there were a few (1.5%) individuals with self‐reported symptoms resembling those of angina.

These results suggest that screening for coronary atherosclerosis can be performed with only self‐reported data and that the screening is only marginally improved by a visit to a health care facility. This corroborates earlier findings, in which other self‐report tools for cardiovascular risk prediction have shown promising results compared with traditional clinical risk assessment tools for the 10‐year risk of cardiovascular death.^11^, ^12^ In addition, 3 main risk groups were identified in our analyses of the population‐ordered distribution of risk: a high‐risk group, a moderate‐risk group, and a low‐risk group. In the high‐risk group (top 30% of the mean absolute risk), up to 65% of all individuals with moderate to severe coronary atherosclerosis can be identified via the self‐report tool (65% of those with SIS ≥4 and 64% of those with CACS ≥100). In contrast, only 3% to 4% of all individuals with moderate to severe coronary atherosclerosis are found in the low‐risk group (bottom 30% of the mean absolute risk).

If our results are recalculated as the number needed to screen, a total of 3.7 CCTA examinations must be performed among high‐risk individuals to detect 1 individual with SIS ≥4 using the self‐report tool (3.5 CCTA for the clinical tool). Further, the proportion of individuals with SIS ≥4 would be around 27% to 28% in the same high‐risk group. Our decision curve analysis showed that both the self‐report and the clinical tool were superior to the PCE at this threshold probability. Results for CACS were similar.

Implementation of a strategy that combines self‐reported risk and subsequent imaging for SIS or CACS would result in the targeted identification of individuals who would benefit from improved lipid‐lowering or other preventive therapies.^1^, ^2^ Future studies will have to test the cost‐effectiveness of such a screening strategy to reduce the overall burden of cardiovascular disease.

We used a combination of data‐driven and manual factor selection to optimize the performance of our tools. The strategy focused on the most easily accessible factors with the greatest impact on model performance. Not surprisingly, information on traditional risk factors,^1^ such as sex, age, lipid‐lowering medication, systolic blood pressure, antihypertensive medication, diabetes, and smoking status, were influential for both the self‐report and the clinical tool. In addition, our data selection and variable importance emphasized the significance of continuous data such as smoking duration and cigarette pack‐years, rather than binary factors such as current smoking, yes/no. It is also noteworthy that many anthropometric measures ranked high in variable importance, confirming the importance of body weight^27^ and abdominal obesity.^28^ Weight at age 20, previously linked to an elevated risk of coronary atherosclerosis,^29^ was one of the most important nontraditional factors, surpassing the impact of weight at the time of examination. On the other hand, a parent's history of myocardial infarction before age 60, ranked low in variable importance. Family history of myocardial infarction is included in some,^30^, ^31^ but not all,^1^ of the previous clinical risk algorithms. We were reassured that the data reduction was successful, given that a tool using all 127 available factors achieved an AUC similar to the clinical tool.

Interestingly, the tools performed significantly better in women than in men. There are numerous reported sex differences that could explain this finding. A recent report from Korea^32^ used similar machine learning methodologies as in the current paper and reported an increased prediction accuracy for IHD in women potentially explained by sex‐specific interaction with risk factors. They suggest that age and waist is more tightly linked to IHD in women and, on the contrary, in men, cholesterol fractions appear most important for prediction.

Using data from the LifeLines imaging trial (ImaLife), Ties and coworkers^33^ tested a prescreening strategy to identify individuals with a high probability of having CACS ≥100. They showed that using a risk algorithm (Systematic Coronary Risk Evaluation 2), primarily developed to identify the 10‐year risk of developing a cardiovascular event, was not very efficient to identify individuals with CACS ≥100. If one or more traditional risk factors were present, around 89% of all individuals with CACS ≥100 were identified; however, using that strategy, around 70% of the population had to be imaged. Our combination of self‐reported risk factors into a risk assessment tool appears to be slightly more efficient, as we could identify 96% of all individuals with CACS ≥100 with the same percentage of population imaging.

### Limitations

A number of limitations of the current study must be acknowledged. First, all population‐based studies suffer from recruitment bias, often resulting in a lower cardiovascular risk level compared with the background population. This is also true in SCAPIS^20^, ^34^ and, therefore, we tested whether recruitment bias could have affected the results by stratifying our sample by several socio‐economic factors. However, our tools performed equally well in the different subgroups, suggesting that recruitment bias does not affect the performance of the tools to a major extent. Second, the derivation and validation populations were mainly of northern European descent and the results may not be generalizable to other geographical regions. Third, we mainly used traditional risk factors as predictors in our tools. It is possible that nontraditional risk factors derived from further biochemical or genetic analyses could aid prediction beyond what was presented here. Fourth, our self‐report tool was based on data acquired at the test sites in SCAPIS and not from home. Truly self‐reported data may introduce more inaccurate measurements and lower the performance of the tools. It is likely that self‐report of anthropometric factors, especially waist and hip circumference, would limit the self‐report model in its current form. Tools developed with fewer factors, selecting the most robust variables, would be important to test in the future. Fifth, we chose SIS and CACS as our outcomes because they are robust and easily accessible measures of coronary atherosclerosis. In the future, we may identify other phenotypes of coronary atherosclerosis (ie, vulnerable forms of atherosclerosis) that confer a higher risk of cardiovascular disease. Furthermore, SIS ≥4 and CACS ≥100 may not be equivalent measures. These cutoffs were chosen to create similar sized groups with coronary atherosclerosis. Sixth, our external validation relied on a pilot trial to the main study. Although using unique individuals and a fundamentally different study protocol, this may affect the generalizability of our findings to broader populations. Seven, it is known from the CARDIA (Coronary Artery Risk Development in Young Adults) study that a positive CAC in early age increases risk of CHD events before the age of 50.^35^ Individuals with IHD before age 50 are excluded from our analyses and our tool is thus limited to the age interval studied.

### Clinical Implications

The presence of coronary atherosclerosis confers an increased risk of future myocardial infarction. Here, we present a self‐report tool, trained and validated in populations without history of previous IHD, which effectively identified individuals with moderate to severe coronary atherosclerosis using self‐reported data alone.

How might a self‐reported risk score be implemented in practice? It is essential to identify high‐risk individuals before they develop events, however, no cost‐effective way of doing this has been presented. In the current report we show that groups of individuals with a high risk of having moderate–severe coronary atherosclerosis can be identified at a low cost. A possible future strategy could be to invite the high‐risk group (top 3 deciles in Table 3 or Table S8) to a clinical visit including a CCTA or CT for assessment of coronary atherosclerosis. If our tool works as described, this will identify as many as two thirds of all individuals with moderate–severe coronary atherosclerosis. It is then likely that general treatment decisions on preventive medication (ie, statins) should be guided by the observed extent of coronary atherosclerosis rather than a composite risk score. Other risk factors detected will inform on individualized interventions. The value of using CCTA (to measure SIS) or CT (to measure CACS) will have to be decided in future studies. However, already from the current data we see that more than 10% of the population will be excluded from imaging due to allergies, poor renal function and issues with image quality if we were to choose CCTA in a screening program. A screening program would eventually have to be tested as a randomized controlled trial to establish evidence for the effectiveness of imaging in primary prevention of cardiovascular disease.

It is also possible that this tool can be effective in the communication with individuals about behavioral changes, given that sharing of medical imaging information has been shown to affect smoking, eating, and physical activity behaviors.^36^

## CONCLUSIONS

We have developed a prediction tool based only on self‐reported data that with good‐to‐excellent discriminative ability can identify individuals with moderate to severe coronary atherosclerosis. The tool performed equally well to a model also including clinical data generated after a clinical visit. The self‐report tool could be the starting point for a screening program to identify high‐risk individuals in need of imaging and further risk evaluation.

## Sources of Funding

This study received funding from: Swedish Heart‐Lung Foundation, Knut and Alice Wallenberg Foundation, Swedish Research Council and VINNOVA (Sweden's Innovation agency), University of Gothenburg and Sahlgrenska University Hospital, Karolinska Institutet and Stockholm County Council, Linköping University and University Hospital, Lund University and Skåne University Hospital, Umeå University and University Hospital, Uppsala University and University Hospital. Work by GB was supported by Heart and Lung Foundation (20210383) the Swedish Research Council (2019‐01140) and LUA/ALF: ALFGBG‐718851 (Agreement on Medical Training and Research).

## Disclosures

E. Hagström reports grants from Pfzier and Amgen (payments to institution), and reimbursements from Amgen, NovoNordisk, Amarin, Pfizer, Sanofi, and Novartis, not related to this article. The remaining authors have no disclosures to report.

## Supporting information

Data S1
Tables S1–S9
Figures S1–S12
Reference [37]
