# Supplementary material for: Self‐Report Tool for Identification of Individuals With Coronary Atherosclerosis: The Swedish CardioPulmonary BioImage Study
Source: J Am Heart Assoc. 2024 Jul 3;13(14):e034603. doi: 10.1161/JAHA.124.034603 (PMC11292769; doi:10.1161/JAHA.124.034603)

Supplemental Material

Data S1.

Supplemental Methods

Cardiac image analyses

A plaque was defined as any tissue structure >1 mm^2 that existed within the coronary artery lumen or close to the coronary artery lumen. We calculated segment involvement score (SIS) as follows: each segment was scored individually as 0 or 1, based on the presence of plaques, irrespective of the degree of stenosis. The sum of all involved segments was then calculated for each patient (ranging from 0 to 17, segments 4 and 15 being mutually exclusive). (8) We calculated CAC score (CACS) using the method described by Agatston. (21)

Rational for selection of cut-offs for outcome

Several studies have confirmed that subclinical coronary atherosclerosis, expressed as the segment involvement score (SIS), is an effective predictor of IHD events, independent of traditional risk factors. (7,8) The meta-analysis by Ayoub (8) indicated a relevant clinical cut-off between 3 and 5 segments to identify a high-risk group. In addition, four other studies that were not included in the meta-analysis, since they did not use SIS as a continuous variable, also suggested an incremental risk of cardiovascular outcomes between 3 and 5 segments involved. (7,17,18) Based on these studies, we chose SIS ≥4 as a cut-off to create a group of individuals at high risk of future IHD. In SCAPIS, we recently showed that SIS ≥4 was found in 11.9% of the population who were aged between 50 and 64 years and who were free of previous cardiovascular ischemic heart disease. (13) Similarly, we chose CACS ≥100 as a secondary outcome to create a high-risk group that would benefit from statin treatment. (2) A cut-off at CACS ≥100 also resulted in similar sized groups with coronary atherosclerosis. The prevalence of CACS ≥100 in SCAPIS was 11.6%. (13)

Data reduction

We first used the Boruta algorithm (36), a machine-learning feature selection algorithm to reduce the number of factors most relevant for identifying SIS ≥4 and CACS ≥100 using the self-report or clinical model. The factors confirmed by Boruta are all relevant to the outcome; however, there is no sharp delineation between confirmed and rejected factors and the algorithm does not weigh in the accessibility of each factor (e.g. data from spirometry may be considered equally relevant to easily accessible questionnaire data). Therefore, the results from the Boruta algorithm were discussed among the lead authors to determine the usefulness of each factor in a possible future clinical screening program as well as if

redundancies could be eliminated and other factors added. In sensitivity analyses, we developed a tool solely based on factors confirmed by the Boruta algorithm and a tool in which all 105 and 127 factors were included without prior feature selection.

Supplemental Results

Factors selected for developing clinical and self-report prediction tools.

The Boruta algorithm confirmed a total of 12 and 19 factors relevant for either SIS ≥4 or CACS ≥100 in the self-reported tool and the clinical tool respectively (Supplementary Table S1). After discussion among lead authors, the data derived from spirometry, accelerometry and a complex multi-question score on mental health was removed. Plasma glycated hemoglobin (HbA1C) concentrations were confirmed by the Boruta algorithm and, therefore, plasma glucose concentrations were added. Systolic blood pressure was confirmed by the Boruta algorithm, which called for a blood pressure measurement and made it possible to add diastolic blood pressure and heart rate to the list of factors without the additional cost of examinations. Several anthropometric measures were confirmed by the Boruta algorithm and, therefore, hip measurement and a question on body weight at age 20 were added. Plasma triglycerides and cholesterol concentrations were confirmed by the Boruta algorithm and, therefore, HDL-C concentrations were added. Questions on family history of myocardial infarction were not confirmed by the Boruta algorithm but added by the lead authors since it scored among the highest among the rejected factors. After the combined data-driven and manual data reduction (see Data S1 for details), 23 factors were available for the clinical tool and 14 factors were available for the self-reported tool (Table S1).

Table S1. Factors used for derivation of assessment tools. Summary of missing data, available factors and factor selection for tool derivation, SIS ≥4.

Factor	Self-report tool		Clinical tool		Missing
	Available	Final	Available	Final	
How often do you have a drink containing alcohol (last 12 months)?	X		X		666 (2.6%)
Number of drinks on a typical drinking day?	X		X		804 (3.2%)
Body height	X	X*†	X	X*†	0
Body weight	X	X*†	X	X*†	0
Hip circumference	X	X†	X	X†	12 (0%)
Waist circumference	X	X*†	X	X*†	8 (0%)
What was your approximate weight when you were age 20?	X	X†	X	X†	1917(7.6%)
Heart rate			X	X†	9 (0%)
Ankle-brachial index, left leg			X*		89 (0.4%)
Ankle-brachial index, right leg			X		102 (0.4%)
Diastolic blood pressure, mean brachial			X	X†	6 (0%)
Systolic blood pressure, mean brachial			X	X*†	4 (0%)
Blood pressure-lowering drugs, self-reported	X	X*†	X	X*†	825 (3.3%)
Hypertension, physician diagnosed, self-reported	X	X*†	X	X*†	815 (3.2%)
Aortic intervention	X		X		815 (3.2%)
Atrial fibrillation	X		X		815 (3.2%)
During the last 2 weeks, have you taken any medication for angina pectoris?	X		X		817 (3.2%)
During the last 2 weeks, have you taken any medication for atrial fibrillation (including warfarin/other anticoagulant)?	X		X		815 (3.2%)
During the last 2 weeks, have you taken any medication for heart failure?	X		X		818 (3.2%)
Have you ever been diagnosed with or been surgically treated for angina pectoris?	X		X		815 (3.2%)
Heart failure	X		X		815 (3.2%)
Intervention of arterial stenosis other than coronary artery stenosis	X		X		815 (3.2%)
Stroke (ischemic or hemorrhagic)	X		X		815 (3.2%)
Chest wheezing in the last 12 months	X		X		922 (3.7%)
Chronic cough	X		X		766 (3%)
Coughing phlegm, when not having a cold	X		X		856 (3.4%)
Coughing when not having a cold?	X		X		766 (3%)
Chronic bronchitis	X		X		856 (3.4%)
Years of coughing phlegm, when not having a cold	X		X		1101 (4.4%)
Years of coughing when not having a cold	X		X		1309 (5.2%)
Age at study visit	X	X*†	X	X*†	0
Sex	X	X*†	X	X*†	0

Glucose			X	X†	2141 (8.5%)
HbA1C			X	X*†	83 (0.3%)
Diabetes diagnosis (derived)	X		X		29 (0.1%)
Duration of diabetes	X	X*†	X	X*†	865 (3.4%) ‡
Duration of insulin treatment	X		X		825 (3.3) ‡
At what age were you diagnosed with diabetes?	X		X		865 (3.4%)
Diabetes (self-reported)	X		X		815 (3.2%)
During the last 2 weeks, have you taken any medication for diabetes?	X		X		825 (3.3%)
How do you treat your diabetes?	X		X		829 (3.3%)
Father's biological figure at 40 years of age	X		X		597 (2.4%)
Mother's biological figure at 40 years of age	X		X		597 (2.4%)
Parental heredity for myocardial infarction	X	X†	X	X†	596 (2.4%)
Parental heredity for stroke	X		X		596 (2.4%)
Hb			X		110 (0.4%)
High- sensitive CRP			X		36 (0.1%)
Cancer medication last 2 weeks	X		X		818 (3.2%)
Have you been prescribed a special diet for celiac disease?	X		X		819 (3.3%)
Have you been prescribed medication for Crohn's disease or ulcerative colitis?	X		X		820 (3.3%)
Have you been prescribed medication for rheumatic disease?	X		X		836 (3.3%)
Were you ever diagnosed with a rheumatic disease?	X		X		815 (3.2%)
Were you ever diagnosed with cancer?	X		X		815 (3.2%)
Were you ever diagnosed with celiac disease?	X		X		815 (3.2%)
Were you ever diagnosed with Crohn's disease or ulcerative colitis?	X		X		815 (3.2%)
Creatinine			X	X*†	21 (0.1%)
HDL			X	X†	43 (0.2%)
TG			X	X*†	36 (0.1%)
Total Cholesterol			X	X*†	37 (0.1%)
Treatment lipids/cholesterol	X	X*†	X	X*†	827 (3.3%)
Physician-diagnosed COPD, chronic bronchitis or emphysema	X		X		815 (3.2%)
Medication for COPD, Chronic bronchitis or emphysema, last two weeks	X		X		817 (3.2%)
FEV1/FVC after bronchodilatation			X		175 (0.7%)
FEV1/VCmax after bronchodilatation			X		170 (0.7%)
Forced expiratory volume in one second post-bronchodilation			X		169 (0.7%)
Forced vital capacity post-bronchodilation.			X		174 (0.7%)
Maximal Expiratory Flow (MEF) 50			X	X*	175 (0.7%)
Slow vital capacity post-bronchodilation.			X		302 (1.2%)
How much do you move in your leisure time (mostly still (tv/reading) to heavy exercise)	X		X		716 (2.8%)

How often training in training clothes in the last 3 months	X		X		764 (3%)
Low-intensity physical activity, % of wear time			X		854 (3.4%)
Moderate- and vigorous-intensity physical activity, % of wear time			X	X*	854 (3.4%)
Quality of sleep under usual circumstances	X		X		826 (3.3%)
Sedentary, % of wear time			X		854 (3.4%)
Time sedentary	X		X		
Describe your work (light to very heavy)	X		X		2724 (10.8%)
Stress (feeling tense, irritable, anxious or having sleeping difficulties as a result of conditions at work or at home)	X		X		861 (3.4%)
Depression index	X		X		1183 (4,7%)
Life events index	X		X		920 (3,7%)
Stress index Bobak			X		1008 (4%)
Work stress index, Copenhagen Psychosocial Questionnaire			X		2204 (8,8%)
Short Form (SF) -12 Mental scale		X*	X	X*	802 (3,2%)
SF-12 Physical scale			X		802 (3,2%)
Sleep apnea	X		X		815 (3.2%)
Sleep apnea, treatment	X		X		831 (3.3%)
Frequency of loud snoring (according to self or others)	X		X		1238 (4.9%)
Frequency of reflux after going to bed	X		X		919 (3.6%)
Hours of sleep per night under usual circumstances	X		X		898 (3.6%)
Respiratory arrest	X		X		955 (3,8%)
Sleep index, Basic Nordic Sleep Questionnaire			X		869 (3,5%)
Tired during daytime	X		X		955 (3.8%)
How many people, including yourself, are there in your household?	X		X		1164 (4.6%)
Social network, the Availability of Attachment (AVAT) index			X		1024 (4,1%)
Social network, the Availability of Social Integration, (AVSI) index			X		988 (3,9%)
Are you born in Sweden?	X		X		564 (2.2%)
Can you raise 20,000 Swedish kronor in a week?	X		X		1059 (4.2%)
In what type of residence do you live?	X		X		571 (2.3%)
Problems to cope with running costs.	X		X		710 (2.8%)
With whom do you share your house/apartment? no one	X		X		577 (2.3%)
With whom do you share your house/apartment? child/children	X		X		577 (2.3%)
With whom do you share your house/apartment? other adult(s)	X		X		577 (2.3%)
With whom do you share your house/apartment? parent(s)/sibling(s)	X		X		577 (2.3%)
With whom do you share your house/apartment? wife/husband/partner	X		X		577 (2.3%)
Early retirement	X		X		650 (2.6%)

Highest education attained	X		X		589 (2.3%)
Sick leave (more than 3 months)	X		X		650 (2.6%)
Unemployed	X		X		650 (2.6%)
Working , part or full time	X		X		650 (2.6%)
Do you get pain in your calves when walking up hills, stairs or hurrying on level ground?	X		X		800 (3.2%)
Do you get pain/discomfort in your chest when walking on level ground at an ordinary pace?	X		X		698 (2.8%)
Do you get pain/discomfort in your chest when walking up hills, stairs or hurrying on level ground?	X		X		752 (3%)
Dyspnea class			X		800 (3.2%)
Mild angina	X		X		752 (3%)
Severe angina	X		X		698 (2.8%)
Are those you are cohabiting with smoking, or have they been smoking, in your home?	X		X		1038 (4.1%)
Current smoking status	X		X		500 (2%)
Average cigarettes during smoking	X		X		500 (2%)
Derived variable for age when the subject started smoking.	X		X		539 (2.1%)
Does the subject smoke occasionally?	X		X		500 (2%)
How many years have you regularly been dwelling in indoor workplaces were people smoke ?	X		X		1143 (4.5%)
How many years have you used snuff?	X		X		1246 (4.9%)
If you stopped using snuff, how much did you use?	X		X		1036 (4.1%)
If you use snuff, how much do you use?	X		X		1044 (4.1%)
Pack years for cigarettes.	X	X*†	X	X*†	856 (3.4%)
Years of smoking.	X	X*†	X	X*†	46 (0.2%)

* Factor selected by Boruta for identifying either SIS ≥4 or CACS ≥100
† Factor selected after discussion among co-authors for identifying SIS ≥4 or CACS ≥100
‡ Missing among individuals with known diabetes.

Table S2. Summary of data in cohort used for tool developed to identify CACS≥100. Clinical characteristics of the 28,701 individuals.

	Total	Female		Male	
	All CACS	CACS < 100	CACS ≥ 100	CACS< 100	CACS ≥ 100
n	28,701	14,049	881	11,205	2566
Sociodemographics					
Age, y	57.5 ± 4.3	57.3 ± 4.3	59.8 ± 3.8	57.0 ± 4.3	59.5 ± 4.1
Education, University degree	12,926 (45.0)	6,965 (49.6)	355 (40.3)	4,657 (41.6)	949 (37.0)
Born in Sweden - yes	24,069 (83.9)	11,694 (83.2)	736 (83.5)	9499 (84.8)	2140 (83.4)
Can get hold of 20 000 SEK* in one week - yes	26,109 (91.0)	12,636 (89.9)	763 (86.6)	10,382 (92.7)	2328 (90.7)
Anthropometry					
Weight at age 20, kg	64.4 ± 12.0	57.0 ± 8.3	56.7 ± 9.6	71.9 ± 2.5	73.2± 11.6
Weight	80.5 ± 15.8	27.5 ± 4.6	28.5 ± 5.1	27.5 ± 3.7	28.5 ± 5.1
Height, cm	172 ± 9.7	165 ± 6.4	163 ± 6.9	178 ± 7.2	179 ± 7.0
Waist circumference, cm	94.2 ± 12.9	89.0 ± 12.3	93.0 ± 13.5	99.0 ± 10.9	102.0. ± 11.6
Hip circumference, cm	103 ± 9.5	103 ± 10.8	195 ± 11.2	103 ± 7.8	103 ± 9.1
Smoking status					
Current smoker	3626 (12.6)	1677 (11.9)	214 (24.3)	1314 (11.7)	421 (16.4)
Former smoker	10,182 (35.5)	5,269 (37.5)	404 (45.9)	3,462 (30.9)	1,047 (40.8)
Duration of smoking, y	21.5± 11.0	21.3 ± 10.9	27.9 ± 12.9	20.7 ± 14.6	23.9 ± 17.0
Treatment					
Cholesterol-lowering medication	1966 (6.9)	672 (4.8)	142 (16.1)	705 (6.3)	447 (17.4)
Antihypertensive medication	5,410 (18,8)	2,304 (16.4)	315 (35.8)	1884 (16.8)	907 (35.3)
Diabetes medication	983 (3.4)	276 (2.0)	68 (7.7)	400(3.6)	239 (9.3)
Diagnosis					
Diagnosed hypertension, n (%)	93 (8.8)	41 (8.1)	6 (19.4)	28 (7.1)	18 (14.4)
Diabetes duration (years)	0.6 (4.1)	0.4 (3.1)	1.7 (8.8)	0.7 (4.4)	0.9 (4.3)
Blood pressure, mmHg					
Systolic	126 ± 17	123 ± 17.6	131 ± 18,3	128 ± 15	133 ± 16
Diastolic	77 ± 10.5	76.4 ± 10.7	79,5 ± 10,8	78 ± 10	80 ± 10
Clinical chemistry					
Total cholesterol, mmol/L	5.5 ± 1.0	5.7 ± 1.0	5.8 ± 1.2	5.4 ± 1.0	5.4 ± 1.1
HDL cholesterol, mmol/L	1.6 ± 0.5	1.8 ± 0.5	1.8 ± 0.5	1.4 ± 0.4	1.4 ± 0.4
LDL, cholesterol, mmol/L	3.5 ± 0.9	3.5 ± 0.9	3.6 ± 1.1	3.5 ± 0.9	3.5 ± 1.0
Triglycerider, mmol/L	1.2 ± 0.8	1.1 ± 0.6	1.3 ± 0.7	1.4 ± 1.0	1.5 ± 1.0
Glucose, mmol/mL	5.7 ± 1.1	5.5 ± 0.9	5.9 ± 1.5	5.9 ± 1.1	6.2 ± 1.5
HbA1c, mmol/mL	36.5 ± 6.3	36.1 ± 5.2	38.6 ± 8.3	36.3 ± 6.3	38.6 ± 9.3
High-sensitive C- reactive protein, mg/L	2.1 ± 4.2	2.1 ± 3.6	2.9 ± 6.3	2.0 ± 4.6	2.4 ± 4.8
Estimated GFR, ml/min/m^2	77.1± 9.7	77.5 ± 9.9	77.8 ± 10.9	76.9 ± 9.2	76.3 ± 10.4
Risk score					
Pooled cohort equation	6.3 ± 5.6	3.3 ± 0.3	5.9 ± 0.5	8.6 ± 0.6	12.6 ± 7.2
Heredity					
Family history of premature myocardial infarction	1694 (5.9)	898 (6.4)	576 (5.1)	576 (5.1)	222 (8.7)
Family history of premature stroke	1876 (6.5)	970 (6.9)	553 (4.9)	553 (4.9)	165 (6.4)
Symptoms					
Angina like symptoms†, %	457 (1.6)	244 (1.7)	26 (3.0)	127 (1.1)	60 (2.3)

SEK: Swedish Krona, LDL: low-density lipoprotein, HDL: high-density lipoprotein, HbA1c: glycated hemoglobin. GFR: glomerular filtration rate. * Equivalent to US$ 1900. †According to the Rose questionnaire.

Table S3. Summary of data in the SCAPIS validation cohort for SIS ≥4. Clinical characteristics of the 872 individuals.

	Total	Female		Male	
		SIS <4	SIS ≥ 4	SIS <4	SIS≥ 4
n	872	391	29	331	121
Sociodemographics					
Age, y	57.7 ± 4.4	57.4 ± 4.2	59.8 ± 4.1	56.9 ± 4.5	59.4 ± 4.3
Education, University degree	324 (37.2)	169 (43.2)	5 (17.2)	114 (34.4)	36 (29.8)
Born in Sweden - yes	611 (70.1)	273 (69.8)	18 (62.1)	232 (70.1)	889(72.7)
Can get hold of 20 000 SEK* in one week - yes	735 (84.3)	327 (83.6)	16 (55.2)	289 (87.3)	103 (85.1)
Anthropometry					
Weight at age 20, kg	65.0 ± 12.0	57.3 ± 8.5	56.7 ± 9,4	71.7 ± 9.3	73.4 ± 11.6
Weight	80.5 ± 14.8	72.5 ± 12.6	75 ± 14.6	87.1 ± 11.9	90.1 ± 14.6
Height, cm	172 ± 9.7	165 ± 6.4	163 ± 7.0	179 ± 7.3	179 ± 6.9
Waist circumference, cm	94.2 ± 12.9	89.0 ± 12.3	93.0 ± 13.5	99.0 ± 10.9	102.0. ± 11.6
Hip circumference, cm	103 ± 8.9	103 ± 10.2	106 ± 11.2	103 ± 6.8	103 ± 9.1
Smoking status					
Current smoker	125 (14.3)	50 (12.8)	7 (24.1)	44 (13.3)	24 (19.8)
Former smoker	343 (39.3)	148 (37.9)	10 (34.5)	131 (39.6)	54 (44.6)
Duration of smoking, y	13.4 ± 15.3	11.8 ± 15.3	22.9 ± 17.9	9.8 ± 14.6	15.1 ± 17.0
Treatment					
Cholesterol-lowering medication	62 (7.1)	20 (5.1)	9 (31.0)	13 (3.9)	20 (16.5)
Antihypertensive medication	181 (20.8)	77 (19.7)	15 (51.7)	49 (14.8)	40 (33.1)
Diabetes medication	20 (2.3)	5 (1.3)	1 (3.4)	6 (1.8)	8 (6.6)
Diagnosis					
Diagnosed hypertension, n (%)	70 (8.0)	29 (7.4)	6 (20.7)	18 (5,4)	17 (14.0)
Diabetes duration (years)	0.4 (3.1)	0.2 (1.5)	1.7 (9.1)	0.3 (2.9)	0.9 (4.3)
Blood pressure, mmHg					
Systolic	124 ± 17	121 ± 16	126 ± 22	125 ± 16	132 ± 18
Diastolic	74 ± 9	71 ± 9	72 ± 9	76 ± 9	78 ± 9
Clinical chemistry					
Total cholesterol, mmol/L	5.8 ± 1.1	5.9 ± 1.0	6.2 ± 1.2	5.7 ± 1.0	5.7 ± 1.1
HDL cholesterol, mmol/L	1.7 ± 0.5	1.9 ± 0.6	1.8 ± 0.6	1.5 ± 0.4	1.5 ± 0.4
LDL cholesterole, mmol/L	3.8 ± 0.9	3.8 ± 1.0	4.1 ± 1.0	3.8 ± 0.9	3.8 ± 1.0
Triglycerider, mmol/L	1.3 ± 0.8	1.2 ± 0.6	1.4 ± 0.9	1.4 ± 0.7	1.6 ± 1.1
Glucose, mmol/mL	5.8 ± 1.0	5.5 ± 0.7	6.0 ± 2.1	5.9 ± 1.0	6.2 ± 1.3
HbA1c, mmol/mL	35.7 ± 5.8	35.3 ± 4.5	36.3 ± 7.7	35.5 ± 5.9	37.6 ± 8.1
High-sensitive C- reactive protein, mg/L	2.2 ± 3.5	2.4 ± 4.2	2.4 ± 2.2	1.8 ± 1.9	2.7 ± 4.8
Estimated GFR, ml/min/ m^2	76.6 ± 9.0	77.1 ± 9.3	76.3 ± 9.9	75.8 ± 8.8	76.8 ± 8.2
Risk score					
Pooled cohort equation	6.3 ± 5.1	3.1 ± 2.4	5.6 ± 3.3	8.2 ± 4.6	11.4 ± 6.0
Heredity					
Family history of premature myocardial infarction	137 (15.7)	71 (18.2)	9 (31.0)	33 (10.0)	24 (19.8)
Family history of premature stroke	45 (5.2)	26 (6.6)	3 (10.3)	12 (3.6)	4 (3.3)

SEK: Swedish Krona, LDL: low-density lipoprotein, HDL: high-density lipoprotein, HbA1c: glycated hemoglobin, GFR: glomerular filtration rate. * Equivalent to US$ 1900.

Table S4. Summary of data in the SCAPIS validation cohort for CACS ≥100. Clinical characteristics of the 1062 individuals.

	Total	Female		Male	
	All CACS	CACS <100	CACS ≥ 100	CACS <100	CACS ≥ 100
n	1062	391	29	331	121
Sociodemographics					
Age, y	57.6 ± 4.4	57.3 ± 4.3	61.2 ± 3.7	57.1 ± 4.4	59.8 ± 4.1
Education, University degree	389 (36.6)	213 (41.7)	3 (10.3)	138 (34.2)	35 (29.4)
Born in Sweden - yes	736 (69.3)	356 (69.7)	15 (51.7)	281 (69.7)	84 (70.6)
Can get hold of 20 000 SEK* in one week - yes	878 (82.7)	417 (81.6)	18 (62.1)	340 (84.4)	103 (86.6)
Anthropometry					
Weight at age 20, kg	64.4 ± 12.0	57.0 ± 8.3	56.7 ± 9.6	71.9 ± 2.5	73.2± 11.6
Weight	80.5 ± 15.8	27.5 ± 4.6	28.5 ± 5.1	27.5 ± 3.7	28.5 ± 5.1
Height, cm	172 ± 9.7	165 ± 6.4	163 ± 6.9	178 ± 7.2	179 ± 7.0
Waist circumference, cm	95.3± 12.9	90.1 ± 12.7	93.5 ± 12.9	99.9 ± 10.0	102.0 ± 13.0
Hip circumference, cm	103 ± 9.5	103 ± 10.8	195 ± 11.2	103 ± 7.8	103 ± 9.1
Smoking status					
Current smoker	158 (14.9)	73 (14.3)	7 (24.1)	52 (12.9)	26 (21.8)
Former smoker	403 (37.9)	180 (35.2)	14 (48.3)	151 (37.5)	58 (48.7
Duration of smoking, y	13.5 ± 15.5	12.7± 15.4	23.0 ± 17.4	12.1 ± 14.4	19.6 ± 16.5
Treatment					
Cholesterol-lowering medication	83 (7.8)	27 (6.7)	7 (24.1)	19 (16.9)	19 (16.0)
Antihypertensive medication	227 (21.4)	70 (17.4)	18 (62.1)	70 (17.4)	45 (37.8)
Diabetes medication	50 (4.7)	13 (2.5)	3 (10.3)	18 (4.5)	16 (13.4)
Diagnosis					
Diagnosed hypertension, n (%)	93 (8.8)	41 (8.1)	6 (19.4)	28 (7.1)	18 (14.4)
Diabetes duration (years)	0.6 (4.1)	0.4 (3.1)	1.7 (8.8)	0.7 (4.4)	0.9 (4.3)
Blood pressure, mmHg					
Systolic	124 ± 17	121 ± 16	125 ± 18	127 ± 17	130 ± 16
Diastolic	71 ± 9	72 ± 9	72 ± 7	76 ± 9	78 ± 8
Clinical chemistry					
Total cholesterol, mmol/L	5.8 ± 1.1	5.9 ± 1.0	6.1 ± 1.2	5.6 ± 1.1	5.5 ± 1.1
HDL cholesterol, mmol/L	1.7 ± 0.5	1.9 ± 0.5	1.9 ± 0.6	1.5 ± 0.4	1.5 ± 0.4
LDL cholesterole, mmol/L	3.8 ± 1.0	3.8 ± 1.0	3.8 ± 1.0	3.8 ± 1.0	3.1 ± 1.0
Triglycerider, mmol/L	1.3 ± 0.8	1.2 ± 0.7	1.5 ± 1.0	1.4 ± 0.9	1.6 ± 1.0
Glucose, mmol/mL	5.9 ± 1.3	5.6 ± 1.1	6.3 ± 3.2	6.0 ± 1.2	6.4 ± 1.5
HbA1c, mmol/mL	36.4 ± 7.4	35.9 ± 5.6	39.0 ± 16.0	36.0 ± 7.0	39.7 ± 10.4
High-sensitive C- reactive protein, mg/L	2.3 ± 3.6	2.4 ± 3.8	2.9 ± 3.2	2.2 ± 3.4	2.3 ± 3.3
Estimated GFR, ml/min/m^2	76.4 ± 9.6	76.8 ± 9.9	77.5 ± 12.6	76.0 ± 8.9	75.8 ± 9.9
Risk score					
Pooled cohort equation	6.3 ± 5.3	3.2 ± 2.4	5.9 ± 3.5	8.5 ± 5.3	11.7 ± 5.9
Heredity					
Family history of premature myocardial infarction	157 (14.8)	84 (16.4)	12 (41.4)	37 (9.2)	24 (20.2)
Family history of premature stroke	56 (5.3)	33 (6.5)	4 (13.8)	14 (3.5)	5 (4.2)

SEK: Swedish Krona, LDL: low-density lipoprotein, HDL: high-density lipoprotein, HbA1c: glycated hemoglobin. GFR: glomerular filtration rate. * Equivalent to US$ 1900.

Table S5. Discrimination of the self-report tool for SIS ≥ 4 by socioeconomic status.

Population size: n = 25,014<br><br>C-statistic (SIS≥4)	Clinical tool
Born in Sweden	0.78 (0.76, 0.78)
Born outside Sweden	0.81 (0.78, 0.81)
University degree -Yes	0.79 (0.76, 0.81)
University degree – No	0.78 (0.76, 0.78)
Can get hold of 20,000 SEK* in one week – YES	0.78 (0.77, 0.78)
Can get hold of 20,000 SEK* in one week – NO	0.79 (0.75, 0.79)

* Equivalent to US$ 1900.

Table S6. Discrimination of the self-report tool for CACS ≥100, by socioeconomic status.

Population size: n = 28,701<br><br>C-statistic (CACS ≥100)	Clinical tool
Born in Sweden	0.78 (0.76, 0.78)
Born outside Sweden	0.77 (0.73, 0.77)
University degree -Yes	0.79 (0.76, 0.79)
University degree - No	0.76 (0.74, 0.76)
Can get hold of 20,000 SEK* in one week - YES	0.77 (0.75, 0.77)
Can get hold of 20,000 SEK* in one week - NO	0.83 (0.78, 0.83)

* Equivalent to US$ 1900.

Table S7. Population-ordered distribution for the clinical tool in identifying SIS≥4. Red - High mean absolute risk (average risk 28.2%); Yellow - Moderate mean absolute risk (average risk 8.8%); Green - Low mean absolute risk (average risk 1.7%).

Decile of risk	Cumulative % of population	Mean absolute risk for SIS≥4 (%)	Number needed to image with CCTA per finding	Cumulative number of CCTAs per finding	Cumulative % of all positives	Age (years)*	Male (%)	SIS (number of segments)*	CACS (Agatston Score)*	PCE (%)
1	10%	38.2	2,6	2.6	30.4	61.7	95.5	3.0	218.1	14.5
2	20%	26.0	3.8	3.1	51.1	60.0	91.7	2.3	126.8	10.9
3	30%	20.3	4.9	3.5	67.3	58.4	87.0	1.8	90.1	8.7
4	40%	12.2	8.2	4.1	77.0	56.9	79.4	1.3	54.5	6.9
5	50%	12.1	8.3	4.6	86.6	56.4	68.6	1.2	32.6	5.5
6	60%	5.9	17.0	5.2	91.3	57.5	41.9	0.8	28.0	4.9
7	70%	4.9	20.4	5.9	95.2	57.4	27.5	0.7	20.6	3.9
8	80%	4.4	22.7	6.4	98.7	57.1	8.9	0.6	15.8	3.0
9	90%	0.6	167	7.2	99.2	55.4	1.4	0.3	5.2	2.0
10	100%	1.0	100	8.0	100	53.5	0.2	0.2	5.1	1.2

SIS, Segment Involvement Score; CCTA, Coronary Computed Tomography Angiography; CACS, Coronary Artery Calcium Score; PCE, Pooled Cohort Equation * mean.

Table S8. Population-ordered distribution for the self-report tool in identifying CACS≥100. Red - High mean absolute risk (average risk 25.5%); Yellow - Moderate mean absolute risk (average risk 8.8%); Green - Low mean absolute risk (average risk 2.6%).

Decile of risk	Cumulative % of population	Mean absolute risk for CACS≥ 100 (%)	Number needed to image with CT per finding	Cumulative number of CTs per finding	Cumulative % of all positives	Age (years)*	Male (%)	SIS (number of segments)*	CACS (Agatston Score)*	PCE (%)
1	10%	36.7	2.7	2.7	30.8	61.8	92.3	2.7	223.8	14.8
2	20%	24.1	4.1	3.3	51.0	60.4	86.5	1.9	108.1	11.5
3	30%	15.6	6.4	3.9	64.0	59.0	78.1	1.4	74.6	9.2
4	40%	13.8	7.3	4.4	75.6	57.4	72.3	1.2	56.9	7.3
5	50%	10.3	9.7	5.0	84.2	57.0	56.5	1.1	43.3	5.8
6	60%	6.0	16.7	5.6	89.3	56.6	49.4	0.7	21.7	4.6
7	70%	5.0	20.0	6.3	93.5	57.2	29.4	0.7	20.4	3.9
8	80%	3.5	28.5	7.0	96.4	57.1	13.8	0.4	13.1	3.0
9	90%	3.3	30.3	7.6	99.2	54.9	2.2	0.4	11.7	2.1
10	100%	1.0	100	8.4	100	52.3	0.0	0.2	4.6	1.2

SIS, Segment Involvement Score; CT, Computed Tomography; CACS, Coronary Artery Calcium Score.; PCE, Pooled Cohort Equation * mean.

Table S9. Population-ordered distribution for the clinical tool in identifying CACS≥100. The first decile presents individuals at the highest mean absolute risk. Red - High mean absolute risk (average risk 26.1%); Yellow - Moderate mean absolute risk (average risk 8.5%); Green - Low mean absolute risk (average risk 2.3%).

Decile of risk	Cumulative % of population	Mean absolute risk for CACS≥ 100(%)	Number needed to image with CT per finding	Cumulative number of CTs per finding	Cumulative % of all positives	Age (years)*	Male (%)	SIS (number of segments)*	CACS (Agatston Score)*	PCE (%)
1	10%	36.6	2.7	2.6	30.6	61.8	92.0	2.7	229.9	16
2	20%	24.4	4.1	3.1	51.1	60.3	86.4	1.9	99.1	11.7
3	30%	17.3	5.8	3.5	65.6	58.9	77.3	1.4	79.2	8.8
4	40%	13.6	7.4	4.1	77.0	57.7	67.5	1.3	57.6	7.2
5	50%	10.3	9.7	4.6	85.6	57.4	51.3	1.0	37.3	5,6
6	60%	6.0	16.7	5.2	90.7	57.1	44.6	0.8	33.6	4.6
7	70%	4.2	23.8	5.9	94.2	56.5	36.6	0.5	15.4	3.7
8	80%	3.3	30.3	6.4	97.0	56.1	18.9	0.4	13.4	2.7
9	90%	1.5	66.6	7.2	98.2	55.1	5.4	0.3	5.6	1.8
10	100%	2.1	47.6	8.0	100	53.0	0.6	0.2	7.2	1.1

SIS, Segment Involvement Score; CT, Computed Tomography; CACS, Coronary Artery Calcium Score.; PCE, Pooled Cohort Equation * mean.

Figure S1. Flow diagram of the validation cohorts included. MI. myocardial infarction; PCI, percutaneous coronary intervention. SIS, Segment Involvement Score. CACS, Coronary Artery Calcification Score

Included in the analysis of SIS

Included in the analysis of CACS

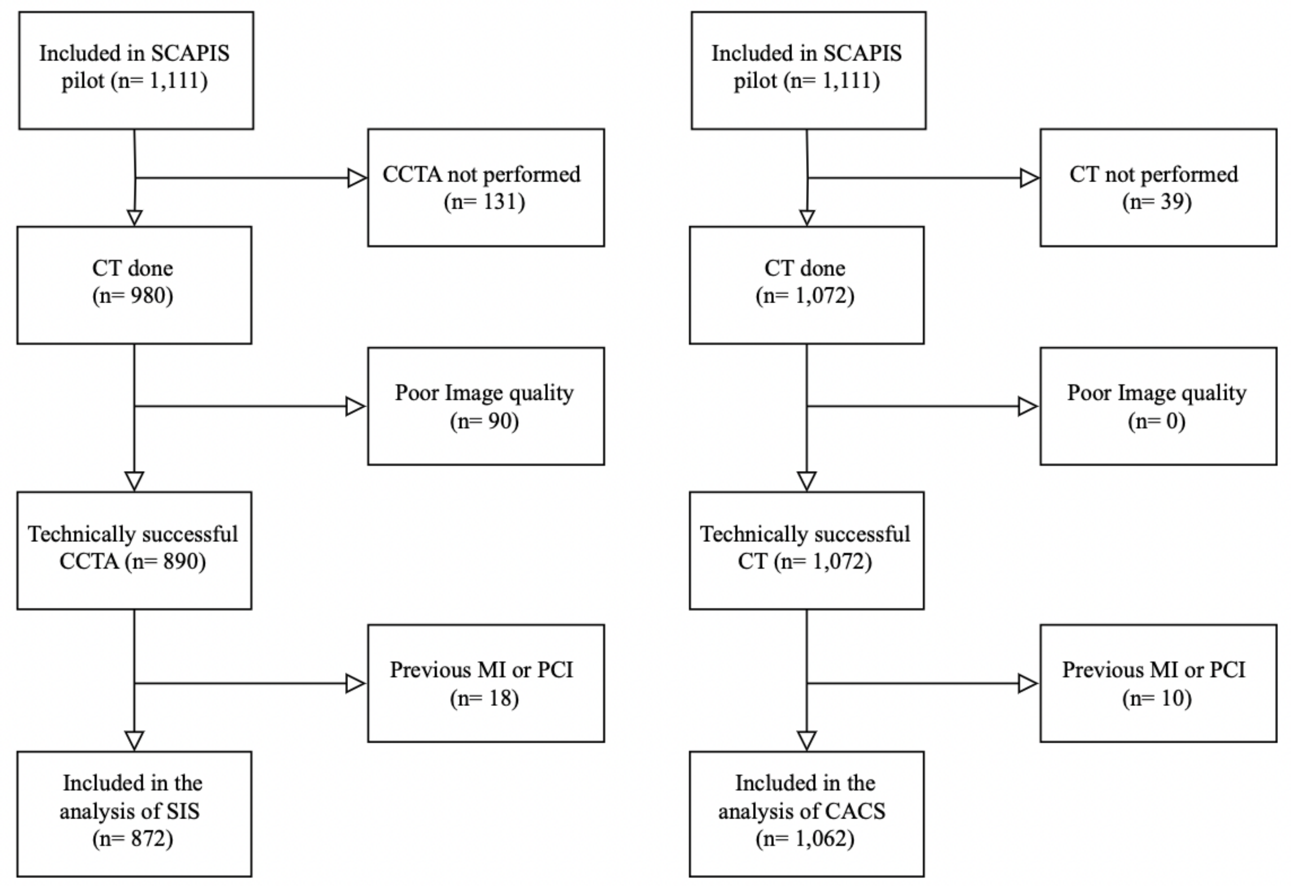

Figure S2. Calibration curves of the self-report and the clinical tools on internal (A, C) and external (B, D) validation set for SIS≥4. The tools show accurate absolute risk prediction across all risk strata

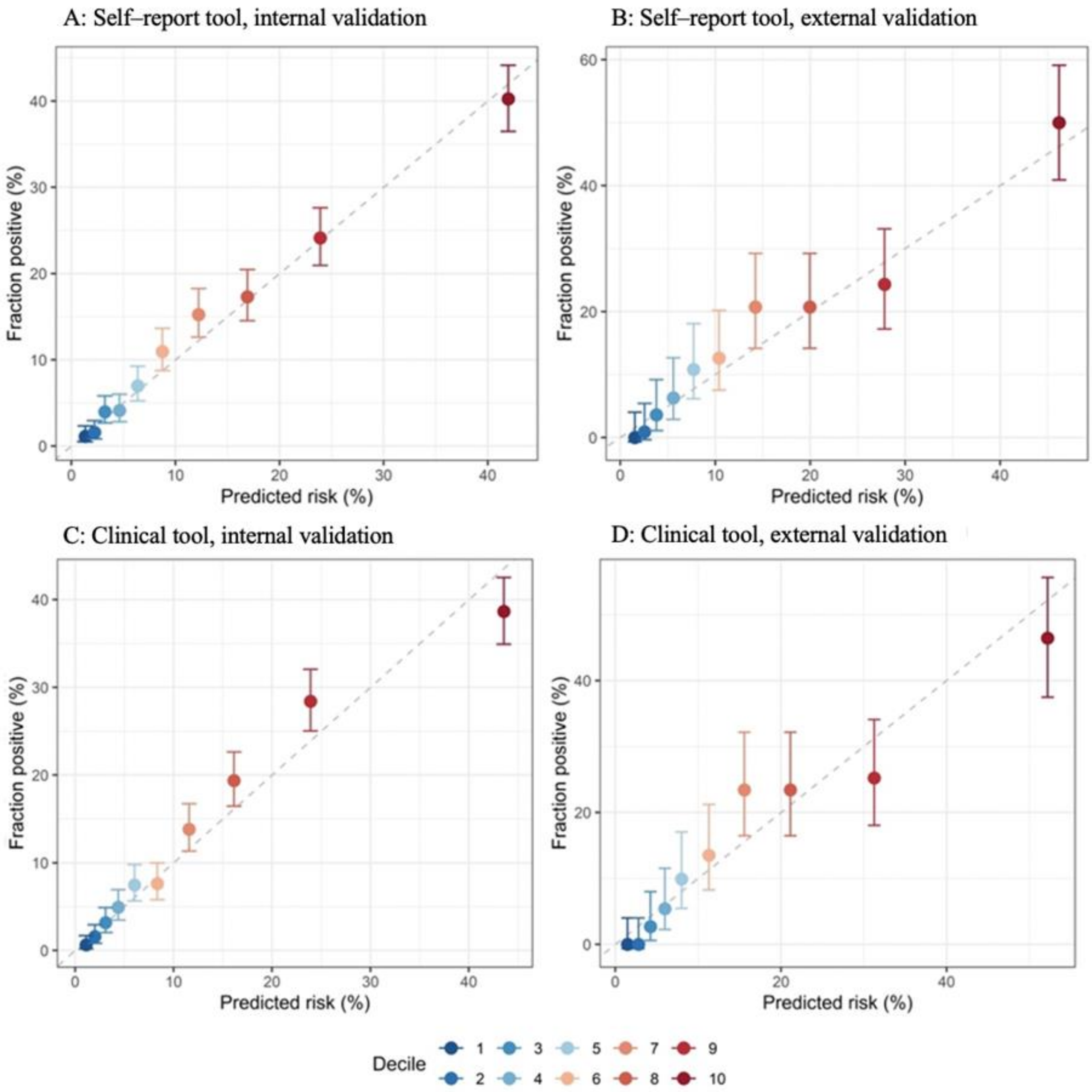

Figure S3. SHAP (Shapley Additive exPlanations) plots for interpretation of each factor's contribution to the self-report tool, for SIS≥4. Males are indicated in purple and females in yellow.

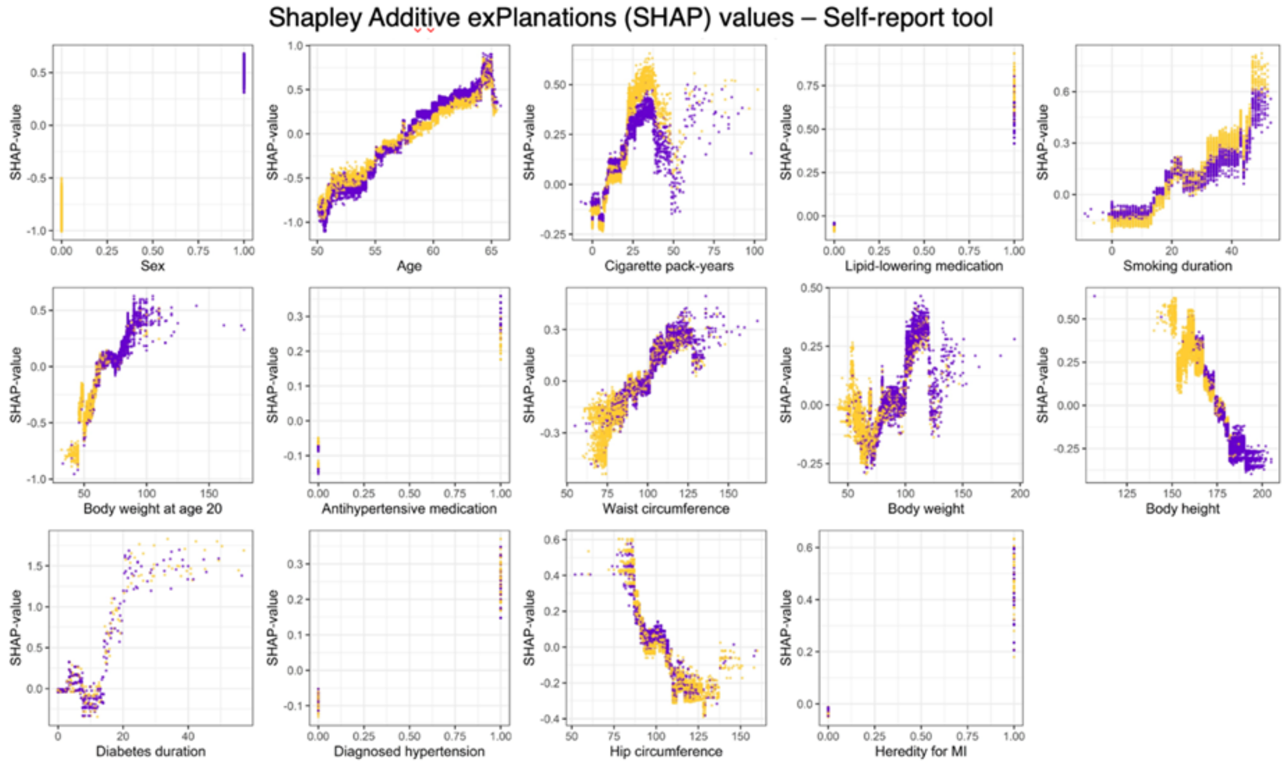

Figure S4. SHAP (Shapley Additive exPlanations) plots for interpretation of each factor's contribution to the clinical tool, for SIS≥4. Males are indicated in purple and females in yellow.

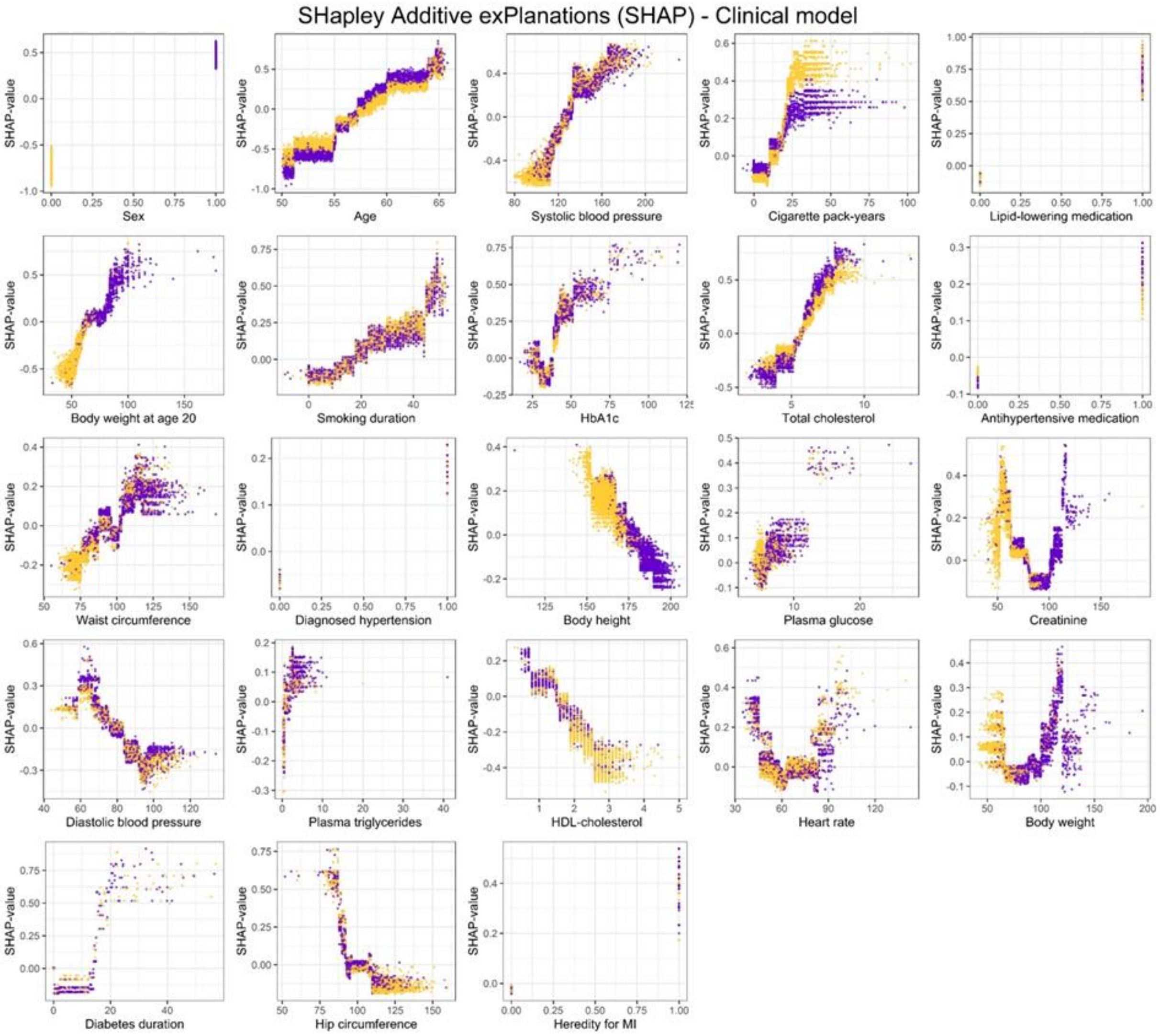

Figure S5. A: Receiver operating characteristic curve for the self-report tool's assessment of CACS≥100 compared to PCE. B: Variable importance of the self-report tool. C: Receiver operating characteristic curve for the clinical tool´s assessment of CACS≥100 compared to PCE. D: Variable importance of the clinical tool.

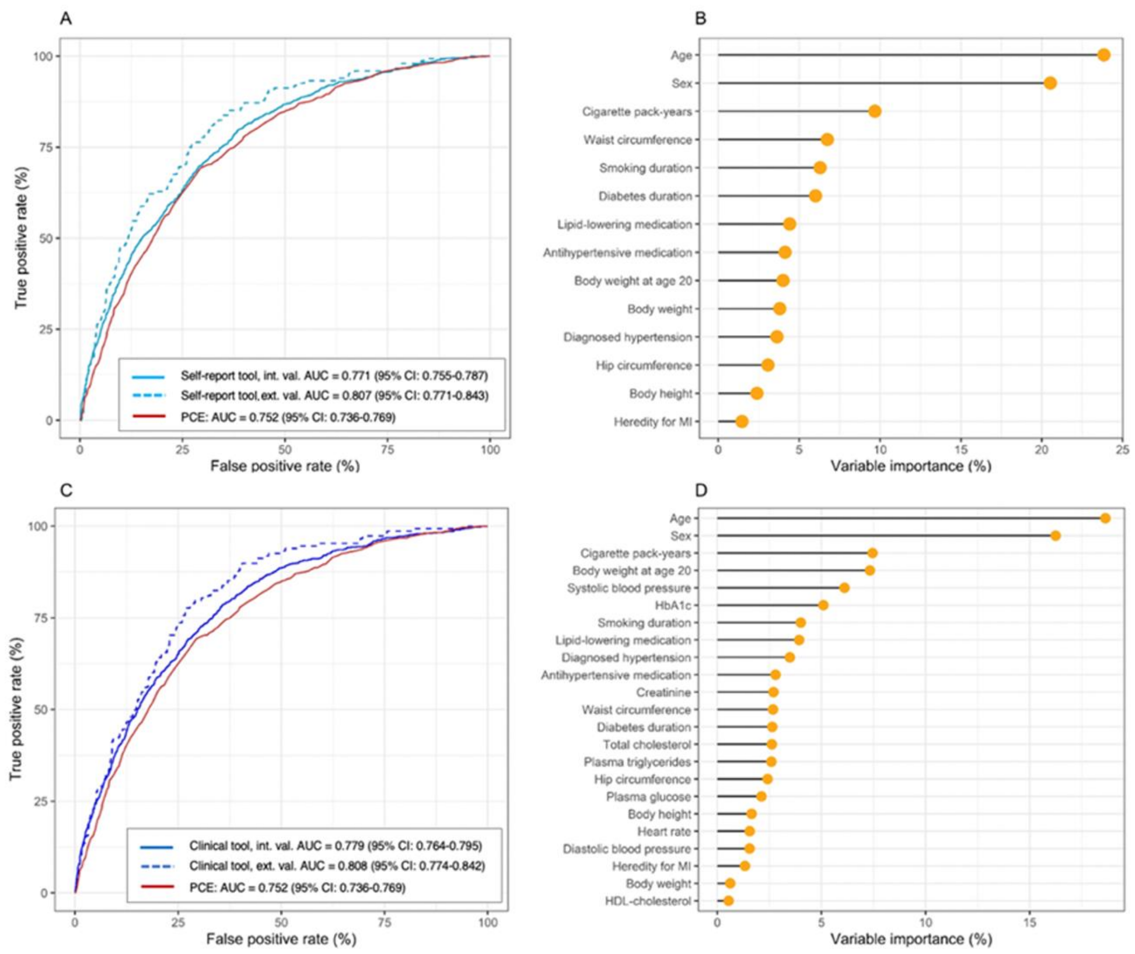

Figure S6. Calibration curves of the self-report and clinical tool on internal (A,C) and external (B,D) validation set for CACS≥100. The tool shows accurate absolute risk assessment across all risk strata

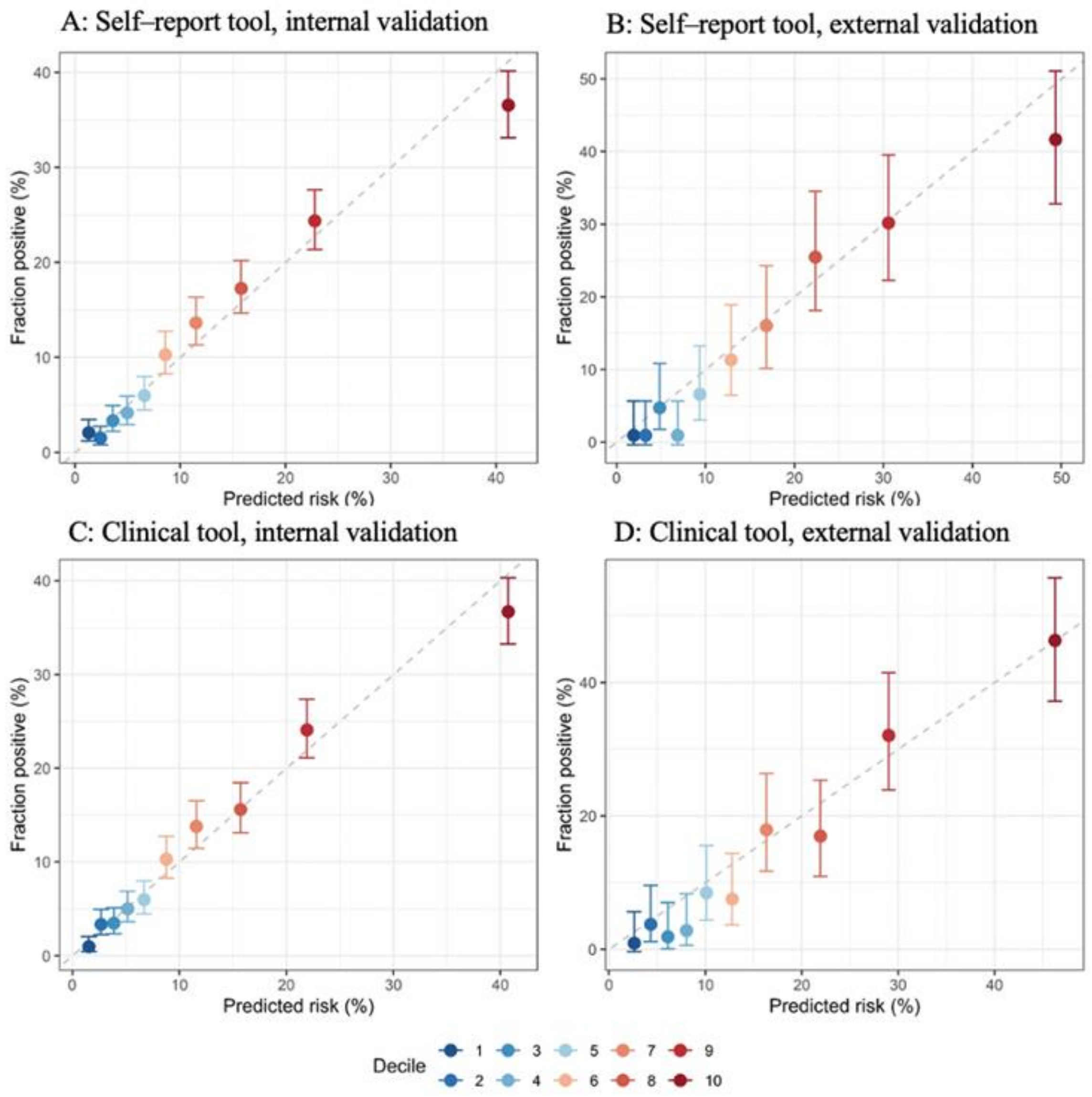

Figure S7. SHAP (Shapley Additive exPlanations) plots for interpretation of each factor’s contribution to the self-report tool, CACS≥ 100.

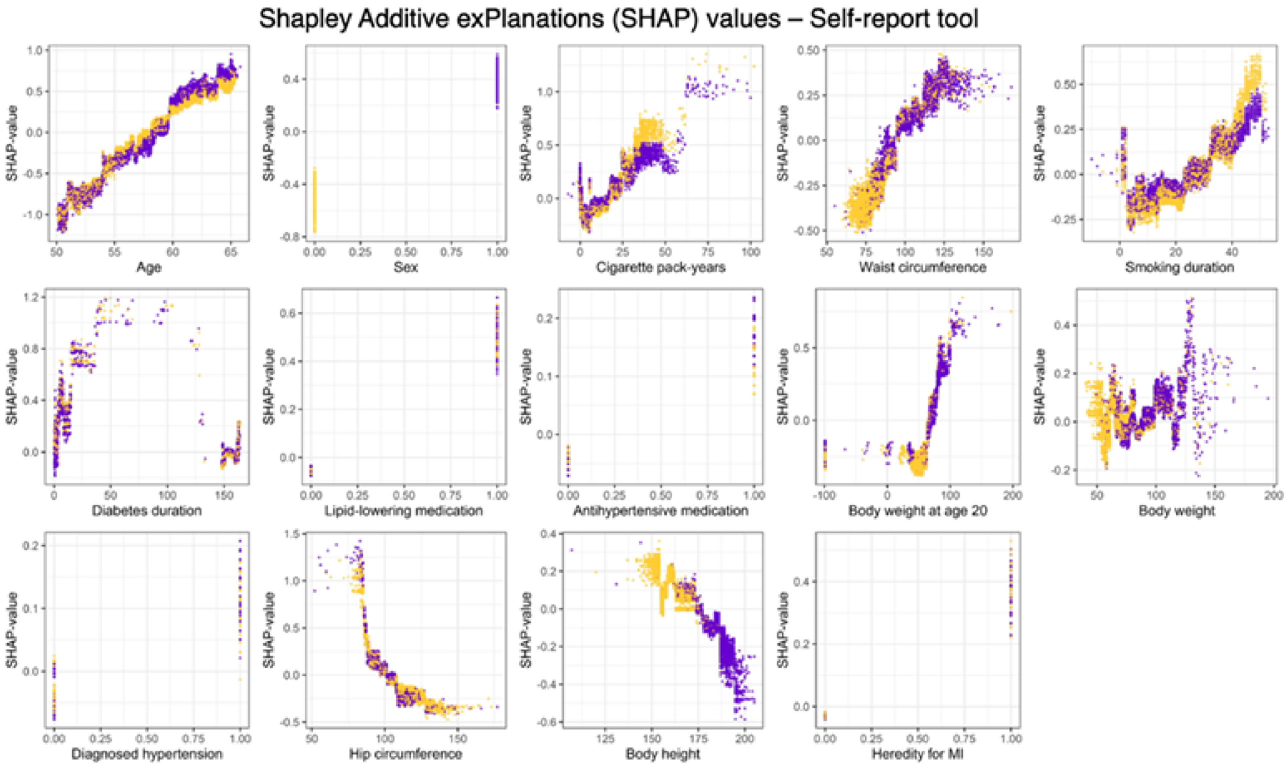

Figure S8. SHAP (Shapley Additive exPlanations) plots for interpretation of each factor's contribution to the clinical tool, CACS≥ 100.

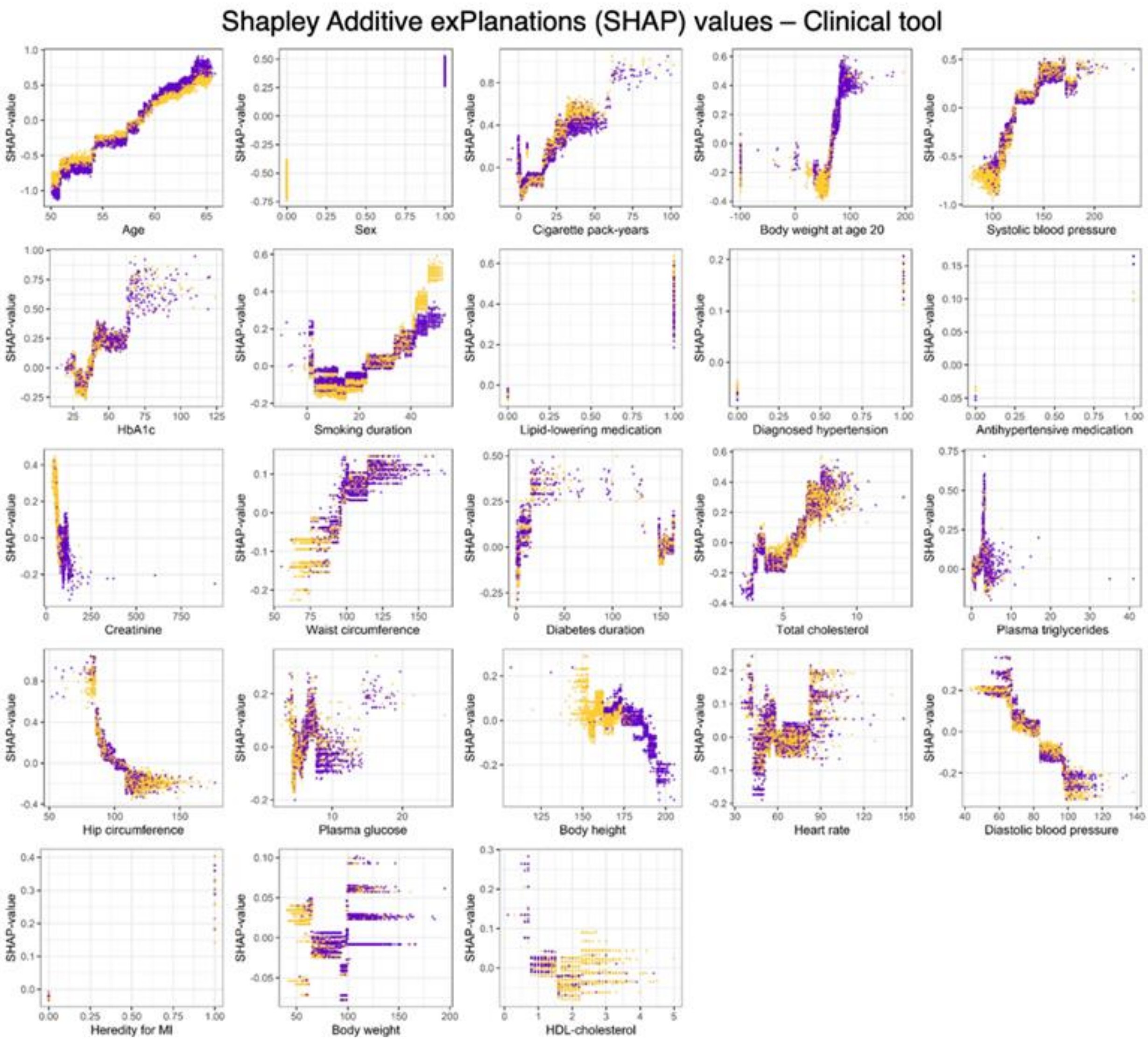

Figure S9. Receiver operating characteristic curves for the self-report tool and the clinical tool assessing CACS≥100 vs. PCE, stratified by age. DeLong results: Age 50-54.9 (NS, $p < 0.05$, 55-59.9 (NS), 60–65.9 ($p < 0.001$).

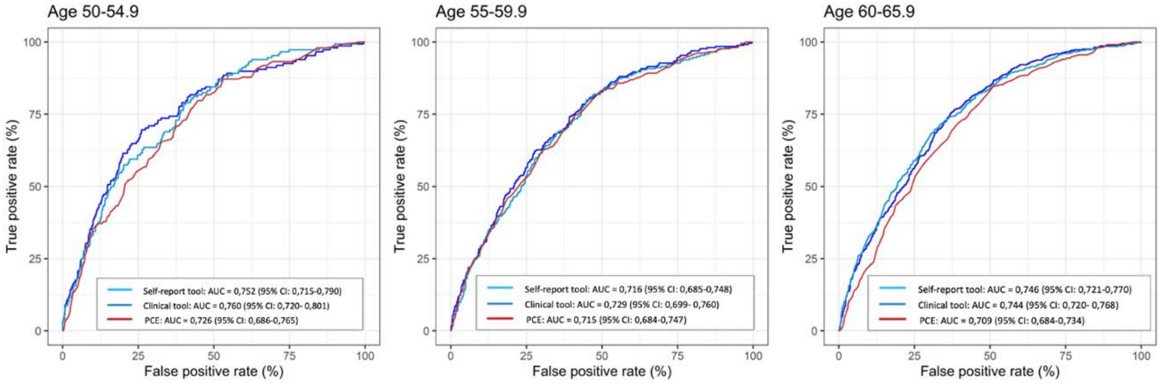

Figure S10. Receiver operating characteristic curves for the self-report tool and the clinical tool assessing CACS≥100 vs. PCE, stratified by sex. DeLong results: Female - Self-report tool vs. PCE ($p < 0.05$), Clinical tool vs. PCE ($p < 0.001$). Male - Self-report tool vs. PCE (NS), Clinical tool vs. PCE ($p < 0.001$).

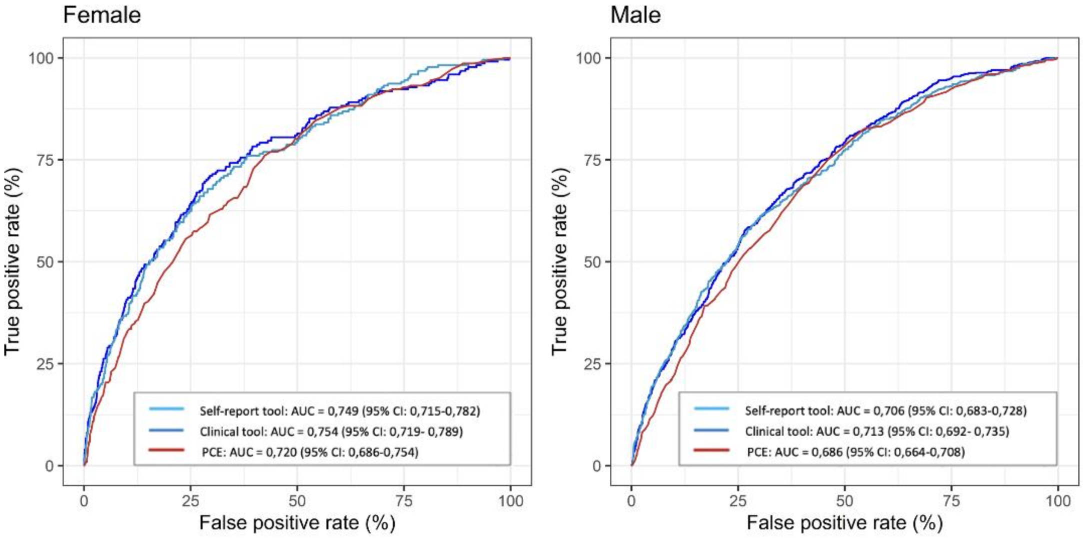

Figure S11. Decision curve analysis graph for the self–report and clinical tool for SIS ≥100

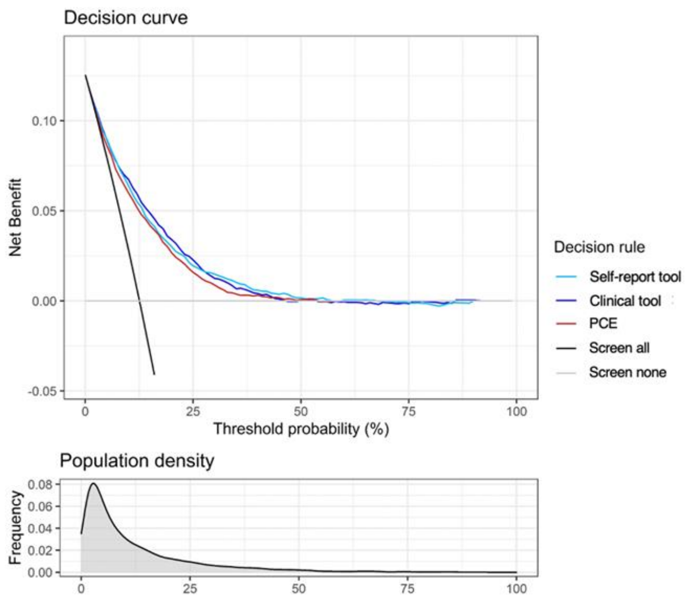

Figure S12. Decision curve analysis graph for the self–report and clinical tool for CACS ≥100

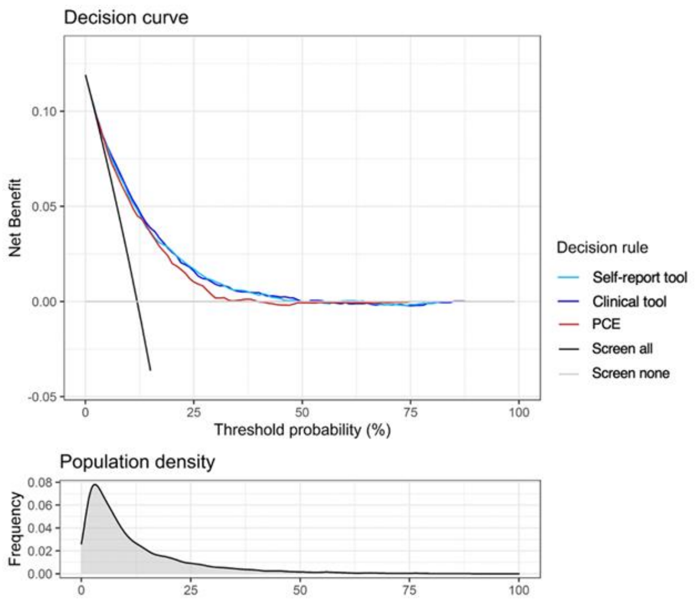

Supplement: Supplementary file 1 — Data S1 Tables S1–S9 Figures S1–S12 Reference [37] [file JAH3-13-e034603-s001.pdf]
